# Enhanced rice leaf disease classification via contour-driven segmentation and optimized deep transfer learning architectures

**DOI:** 10.1371/journal.pone.0348290

**Published:** 2026-05-07

**Authors:** Ummer Shakeel, Muhammad Asif Habib, Muhammad Yasir, Muhammad Umar Chaudhry, Muhammad Munwar Iqbal, Mudassar Ahmad, Qaisar Abbas, Muhammad Abdul Qayum

**Affiliations:** 1 Department of Computer Science, University of Engineering and Technology Taxila, Taxila, Pakistan; 2 College of Computer and Information Sciences, Imam Mohammad Ibn Saud Islamic University (IMSIU), Riyadh, Saudi Arabia; 3 Department of Computer Science, University of Engineering and Technology Lahore, Faisalabad Campus, Pakistan; 4 Department of Computer Engineering, Bahauddin Zakariya University, Multan, Pakistan; 5 Department of Computer Science (DCS), National Textile University (NTU), Faisalabad, Pakistan; Najran University College of Computer Science and Information Systems, SAUDI ARABIA

## Abstract

Pakistan is the fourth-largest rice producer and the fifth-largest exporter worldwide. Timely disease detection remains challenging due to the scale of cultivation and reliance on manual monitoring. Developing reliable, ongoing computerized systems for plant health management is essential for efficient disease control. A deep learning approach is used as the core method to identify diseases in rice leaves. This methodology employs a range of advanced deep learning architectures to achieve top-tier feature extraction and classification. The publicly available rice leaf disease dataset on Zenodo supports research reproducibility and data transparency. We systematically process a balanced dataset of 1914 image samples using Python with TensorFlow and a GPU to enable high-speed computation for large-scale image processing. This study conducts a systematic comparative evaluation of five deep transfer learning architectures (InceptionV3, DenseNet201, ResNet152V2, EfficientNetV2L and MobileNetV2) trained independently. The base backbone models are then integrated with guided GrabCut segmentation with contour-detection method for interpretable disease localization. In this work, the methods of segmentation by GrabCut and contour detection are introduced to make the results of the study easier to interpret and explain the disease areas, but the final classification outcomes are obtained only on the basis of the underlying deep transfer learning models. As a result, infected leaf areas can be identified more effectively, allowing for better understanding and explainable of the disease.To enhance interpretability, GrabCut segmentation and contour detection are applied as post-hoc visualization techniques to highlight diseased regions corresponding to CNN predictions. These techniques do not influence the classification training process. All five models InceptionV3, DenseNet201,ResNet152V2,EfficientNetV2L and MobileNetV2 demonstrated their effectiveness in detecting rice diseases during training, validation, and testing phases, with models trained over 30 epochs. The training methods and accuracy rates of the models were compared during validation and final testing. InceptionV3 demonstrated the most moderate performance of 98.80% training, 98.44% validation, and 98.43% test accuracy, which means that it has strong generalization and consistent learning behavior. The performance of very high-density networks such as DenseNet201 (98.72% train, 98.43% val, 98.43% test), ResNet152V2 (99.02% train, 99.22% val, 97.39% test), EfficientNetV2L model accuracies (39.01% train, 48.70% val, 44.50% test) also showed competitive results, which validated the effectiveness of deep transfer learning in the classification of rice leaf disease, while MobileNetV2 model accuracies (98.09% train, 98.18% val, 96.87% test) indicate that a lightweight model can still achieve reliable classification performance with lower computational complexity. In general, the comparative analysis defines InceptionV3 as the most stable and efficient model in the framework proposed. These results illustrate InceptionV3 superior generalization ability, supported by explainable methods for improved feature localization, confirming the viability of transfer learning for accurate and practical rice disease detection using GrabCut segmentation and contour detection technique. The complete implementation code and data used for the research experimentation is publicly available at https://github.com/ummershakeel03/Rice-Leaf-Diseases-Classification for reproducibility and reuse.

## Introduction

Pakistan is a major rice producer; it ranks as the world’s fourth-largest producer of rice. It is also the fifth-largest rice exporter. Farmers cultivate rice plants, but most lack understanding of how to protect the plants. They often rely solely on their naked eye to detect plant health issues. However, manual monitoring on large farms is challenging, time-consuming, and expensive. Effective disease detection and classification require specialized skills and continuous plant monitoring. Rice is a widely grown crop in many key countries and is a vital global commodity. Its production depends heavily on early detection of diseases [1]. Climate change and global warming have greatly affected agriculture and rice yields. Diseases reduce both the quality and quantity of rice throughout the growing season. In South Asia, lowland rice cultivation experiences an average productivity loss of more than 10% due to diseases. Rice fields are in deplorable condition and may see a drop in yield of up to 45%. It is necessary to use developing technologies to support farmers [2].

To ensure healthy plant growth, farmers must identify and treat diseases early before they cause significant damage; quickly diagnosing and classifying diseases is essential to maximize productivity. Monitoring rice plants requires substantial effort and expertise for accurate identification. Conversely, an automated system is necessary to detect and categorize rice leaf diseases at their early stages. Therefore, an innovative system utilizing image processing technologies is needed to assess plant health. Image processing and computer vision can assist in observing and diagnosing symptoms of diseases on leaves, stems, and fruits [3]. Diseases may appear in various parts of the plant. Additionally, this technology can detect unhealthy symptoms in rice leaves. Disease infections have a detrimental effect on the quality and quantity of crops, ultimately resulting in reduced production in the agriculture sector [4]. Shorten and Khoshgoftaar [5] discovered that data augmentation is often a critical step to enhance deep learning model performance, especially when training data is limited. As a development, Google *InceptionV*3 employs convolutional neural networks to improve image classification efficiency through convolutional factorization and the application of asymmetric convolutions and label smoothing methods [6]. Through these enhancements, the model attains improved efficiency and higher accuracy compared to earlier versions [7]. InceptionV3, a CNN architecture developed by the Google Brain Team, balances performance and computational resource demands. By using Inception modules comprising parallel applications of different-sized filters,Inception networks can learn both fine-grained and coarse-grained features simultaneously. These modules include 1 × 1 convolutions to reduce dimensionality, lowering computational costs and enabling deeper networks. Additionally, *InceptionV*3 employs auxiliary classifiers during training to address the vanishing-gradient problem, enhancing the model’s capacity to extract discriminative features. DenseNet201 is a CNN architecture featuring layers of dense connectivity. It employs 201 layers and dense blocks, which help prevent the vanishing gradient problem and promote feature reuse [8]. This configuration results in better gradient flow, faster convergence, and improved performance with comparable parameters compared to traditional architectures. DenseNet201 has considerable applications in image classification, especially where high accuracy is needed. Refer to the original research for more details on its design and performance [9]. ResNet152V2, an improved version of the ResNet architecture, includes residual learning via identity mappings to tackle the vanishing gradient problem [10,11]. It has 152 layers and uses skip connections, which improve gradient flow during backpropagation. This architecture enables the network to learn deep features efficiently. These models, integrated into frameworks like Keras or MATLAB, come with pre-trained weights, making them suitable for accurate transfer learning applications.

The rice crops in Pakistan are vulnerable to various rice diseases, including viruses, fungi, and bacteria, which can cause significant damage and lead to heavy losses. One of the most critical diseases to address is BLB, or Bacterial Leaf Blight; taking preventative action is essential. Rice in Asia faces a significant challenge due to the rapid spread of diseases during the monsoon season, which lasts from June to September. A key feature of this disease is known as Kresek, caused by bacteria present in the rice nursery. Throughout the progression of the disease, this condition ultimately advances to the leaf blight stage. The disease can cause losses of up to 70% in South Asia. The risk of such losses remains because current knowledge about various disease types, weather variations, and effective management practices is still insufficient. It is widely believed that people in Asia are especially vulnerable to the devastating effects of bacterial leaf blight compared to other regions. This heightened susceptibility is mainly due to the heavy rainfall typical of the monsoon season [12].

Three major infectious threats to global rice cultivation attack rice leaves: Leaf Blast (LB), Brown Spot (BS), and Bacterial Leaf Blight (BLB). The fungus that causes Leaf Blast produces slender lesions that damage leaf tissue. The development of brown circular spots through Brown Spot reduces both crop yield and grain quality. The pathogen spreads quickly through water sources, causing leaf-tip yellowing and subsequent wilting of rice plants [13].

### Brown spot

**Fig 1:**
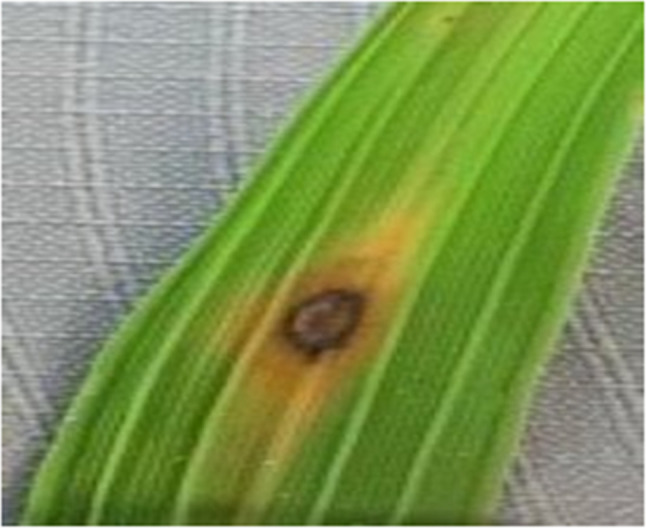
Brown spot [2].

When we consider the group as a whole, Fungus (Cochliobolus miyabeanae) has been one of the major diseases causing yield loss in rice worldwide, particularly among Brown Spot. Disease symptoms include small, round brown spots on leaf blades, which lead to decreased photosynthesis and reduced plant vigor. Yield facts indicate that the disease is often encountered with more symptoms, and understanding how these symptoms relate to each other is complex, as it is affected by factors like exhausted soils and plant stresses. Inadequate control of Brown Spot results in low rice yields, poor quality, and less grain filling [14]. Fig. 1, shows Brown Spot on rice leaves.

### Leaf blast

**Fig 2:**
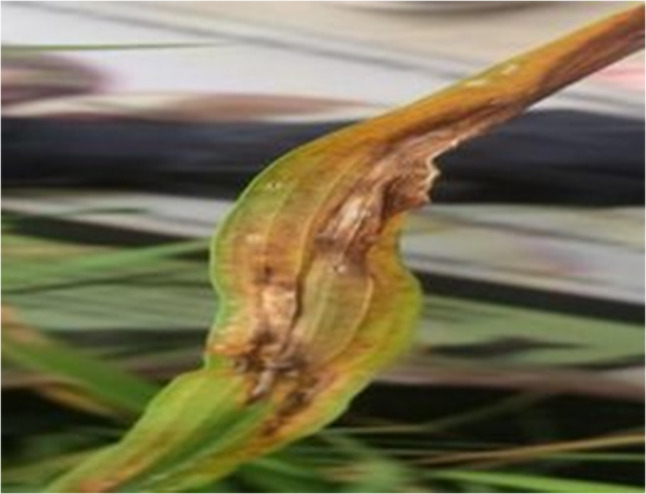
Leaf blast [2].

Another disease, leaf blast, is caused by the fungal pathogen Magnaporthe oryzae. The fungus creates various-sized, dark, rhomboid patterns on the leaf surface, which often merge into large, blotchy areas. Necrotic plant tissue, through which vertical infection spreads, can also damage the growing tip, leading to plant death. The presence of highly susceptible hosts makes this disease a serious problem when it spreads widely in areas with hot, humid conditions. If ’Leaf Blast’ becomes an epidemic in your field, it can aggressively attack rice crops, resulting in significant yield loss [15]. Fig. 2 shows Leaf Blast disease on rice leaves.

### Bacterial leaf blight

**Fig 3:**
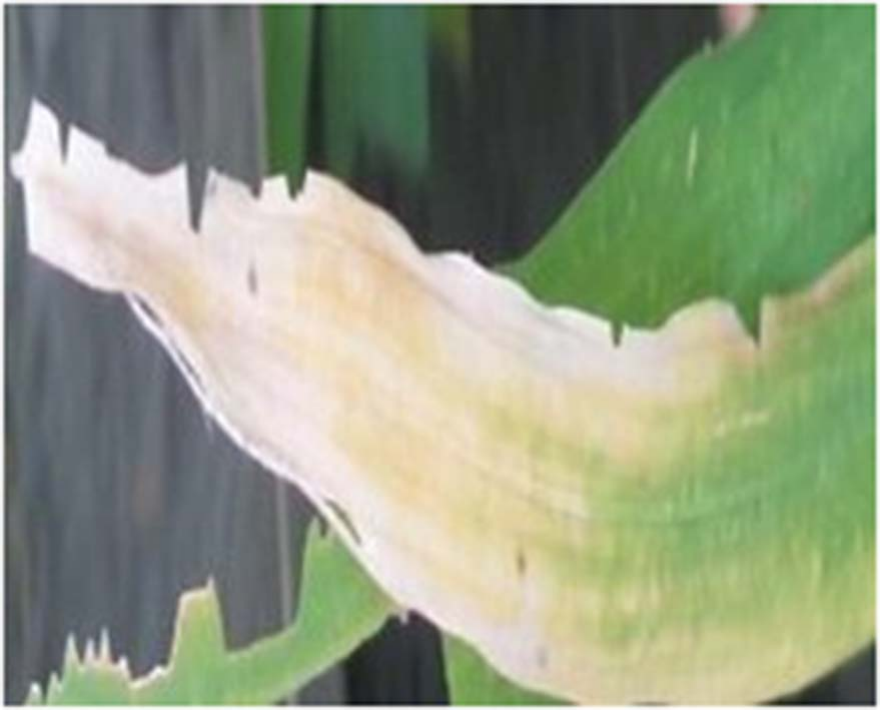
Bacterial leaf blight [2].

Bacterial Leaf Blight of rice is caused by a bacterial pathogen called Xanthomonas oryzae. It begins with foliar symptoms such as water-soaked streaks along the margins of the leaves, and then the affected areas turn yellow, followed by brown, leading to wilting. Symptoms are barely noticeable in damp conditions. The disease proliferates under warm, wet conditions and reduces photosynthesis capacity by half for three weeks after infection, severely restricting growth and yield. Severe infections can be even more destructive in a field, decreasing kernels per head or ear and their weight [16] Fig. 3 shows Bacterial Leaf Blight rice leaf disease.

Regarding image processing, the contour-based technique involves detecting edges, extracting object outlines, determining object boundaries, identifying various objects, and extracting features based on regions. Contour-based detection is a highly effective method for recognizing and evaluating the shapes and boundaries of items in an image. To reduce noise, a Gaussian blur is applied after converting the image to grayscale. A binary threshold then creates a high-contrast image that emphasizes object edges. Contours are continuous curves that connect all points along a boundary and can be identified using techniques like the ‘findContours‘ function in OpenCV. This approach efficiently delineates distinct regions, such as rice leaf lesions, to facilitate further analysis or classification using machine learning algorithms for disease detection. Data augmentation with the Augmentor Python library is also employed. Contour detection techniques can improve the accuracy of rice leaf disease identification, as they are an efficient approach. Initially, images of the leaves are preprocessed into grayscale, and a Gaussian blur is used to reduce noise. Then, a binary threshold highlights the contours of affected regions. These contours are identified using OpenCV’s contour-finding capabilities. Once separated, the affected areas can be expanded and cropped for further analysis with a trained deep learning model, such as InceptionV3. This enables more detailed investigation and classification of diseases affecting rice leaves, including leaf blast, brown spots, and bacterial leaf blight. By applying a contour-based method with pre-trained deep learning models, the process of object analysis and visual pattern recognition is enhanced.

Approximately eight billion people rely on rice farming annually because diseases in rice fields cause financial losses for distributors. The disease assessment system operated by expert specialists functions slowly due to the lack of a comprehensive disease observation system. Transfer learning and image processing techniques are used. The technical framework includes modern analytical methods capable of accurate disease detection. Current market-available AI models assist farmers in detecting diseases early and increasing their productivity. Achieving high accuracy in disease identification depends on the distinct architectural designs of InceptionV3, ResNet152V2, and DenseNet201. Agricultural workers can identify the most affected areas of disease using contour methods to make precise assessments. Rice leaf disease detection systems have become a research priority as farmers depend on them for survival.

Rice leaf diseases pose a significant threat to agricultural production because their detection requires immediate and accurate assessment for sustainable control. Traditional methods, if they can be called that, rely on manual inspection, which is slow, labor-intensive, and prone to human errors. Current systems do not adequately handle various environmental conditions and different disease symptom presentations effectively. Therefore, this work involves employing pre-trained deep-learning models along with contour detection techniques to identify three deadly rice leaf diseases. The three disease categories targeted by this detection system are Brown Spot, Leaf Blast, and Bacterial Blight. Processing field images results in feature extraction that produces classified data through transfer-learning techniques, followed by implementing image processing methods such as GrabCut segmentation and contour detection.

The main goal is to identify rice plant leaf diseases through transfer learning and image processing methods.

- To Implement multiple CNN architectures (InceptionV3, DensNet201, ResNet152V2, EfficientNetV2L, and MobileNetV2) for classification of rice plant diseases.

**Architectural diversity**: Each CNN model addresses specific challenges (e.g., InceptionV3 for multi-scale features, ResNet for depth), ensuring robustness across disease patterns.

- To utilize multiple features from overall CNN layers to enhance model effectiveness through various techniques, simplifying the process.

**Feature fusion**: By aggregating features from all layers, the model captures both low-level (edges) and high-level (textures) patterns, improving classification accuracy.

- After extraction of characteristics from the base models, GrabCut and contour detection techniques are used in image processing to identify areas of rice disease.

**Localization**: GrabCut and contours provide explainability, linking predictions to physical regions in images critical for agricultural diagnostics.

This study addresses the challenge of accurate and interpretable rice leaf disease detection by combining deep transfer learning models with visual localization techniques. Three widely used CNN architectures (InceptionV3, DenseNet201,ResNet152V2, EfficientNetV2L and MobileNetV2) are evaluated for classification, while contour-driven visualization highlights disease regions corresponding to model predictions.

It applies CNN with different architectures (InceptionV3, DenseNet201, ResNet152V2, EfficientNetV2L and MobileNetV2) to identify rice plant diseases by utilizing their superior features, such as multi-scale feature extraction, depth effectiveness, and robustness to analyze comprehensive patterns. It also improves accuracy by synergizing the multi-lying characteristics (catching edges and textures) and using GrabCut with contour identification to localize disease a bridge between predictions and visual symptoms in the field of agriculture. Comparative evaluation of multiple architectures such as InceptionV3 with DenseNet201 and ResNet152V2 to extract data features for rice plant disease classification, enhancing overall model effectiveness. This work advances previous studies that only addressed classification by using CNN-derived features from different layers to boost diagnostic accuracy through simplified procedures. Rice is one of the most important food crops worldwide, and detecting any leaf disease promptly is a significant challenge due to manual surveillance procedures that are limited and rely on human inspection. Modern deep learning methods often lack detailed segmentation techniques, leading to imprecise localization of infected areas. Additionally, publicly available datasets tend to be unbalanced, physiologically inconsistent, and do not accurately reflect real-world conditions, which hinders effective generalization. Although models like InceptionV3, DenseNet201,Resnet152v2,EfficientNetV2L and MobileNetV2 have strong feature extraction abilities, their integration with contour-based segmentation techniques to enhance interpretability has not been thoroughly explored. This highlights the urgent need for a scalable and explainable framework capable of combining these models with complex segmentation methods, such as GrabCut, to enable accurate and interpretable diagnosis of rice leaf diseases.

## Background

The economy of Pakistan is centered on the agricultural, industrial, and trade sectors, with rice as one of the major cash crops alongside cotton, wheat, and maize. Rice is the second most important crop, boosting farmers’ incomes and supporting exports that generate billions of dollars in foreign currency. Pakistan is also the 4^*th*^ biggest exporter of rice and the 11^*th*^ largest producer of rice. Rice is among 3.2% of agricultural value-added and 0.7% of GDP. Two types of rice are grown in the country, and the Basmati rice is very pricey owing to its quality and market demand. During the 2014–15, the rice cultivation area increased by 3.1% to 2.847 million hectares, while production rose by 1.5% to 6.9 million tons. The significance of rice is reflected in exports, which earned 1.848 billion during this period [17]. Rice farming faces ongoing threats from various diseases and destructive insects. Compared to other countries, every nation in South and Southeast Asia reports the highest number of diseases responsible for yield loss [18]. The research findings were obtained through a survey, while the study used a quantitative approach. Government of Punjab, Pakistan, “Pest Warning 6 Quality Control of Pesticides, Agriculture Department.”

Between the years 2014–2017, an extensive survey was done in 18 districts of Punjab, Pakistan such as Sargodha, Faisalabad, Lahore, Gujrat, and Sialkot, where regular checkups were done to observe the Brown Spot (BS) disease. The statistics indicated that Sargodha district had the highest BS disease incidence whereas Okara had the lowest incidence. In addition, Basmati Super was the most susceptible as evidenced by the rate of 51 percent, whereas Basmati 386 was the least susceptible at around 6.5 percent [19].

The highest incidence was observed in the Basmati-growing districts (including Sheikhupura, Gujranwala and Hafizabad) where bacterial leaf blight (BLB) was the most prevalent, and there was increased prevalence in the year 2010–2012 and 2013 but non-Basmati areas reported relatively lower prevalence of the disease was reported as well in the year 2010, 2012 and 2013 [20]. Brown spot and rice blast diseases were also highly affected by the epidemiological factors where frequency of disease outbreak was observed to be closely associated with increasing humidity and rainfall, and the opposite observed with low temperatures.

## Literature review

The successful cultivation of crops heavily depends on the timely identification of plant leaf symptoms, determining the type of pest or disease, and assessing the disease rate. Unfortunately, many agricultural centers still rely on manual detection, requiring farmers to travel long distances for disease diagnosis. In contrast, our proposed system uses automatic image processing to diagnose diseases in rice plants, providing a detailed report with symptoms and treatment recommendations. Scientists used MATLAB image processing systems to evaluate diseases in banana, beans, jackfruit, lemon, mango, potato, sapota, and tomato plants. A genetic algorithm separated plant images through research involving experiments with prediction methods such as ANN, Bayes classifiers, Fuzzy Logic, and hybrid approaches [21]. The proposed article discusses feature extraction methods, segmentation, and classification using k-means clustering, Otsu thresholding, and color co-occurrence [22].

The proposed (CNN) technique for detecting rice leaf diseases achieved an impressive classification accuracy of 95%. To optimize disease pattern learning, the model was trained on a rice leaf image dataset with a batch size of 10 for 25 epochs [23]. The deep learning approach demonstrates its effectiveness in large-scale rice blast disease monitoring, showcasing its suitability for agricultural oversight. The model, trained on a reasonably preprocessed dataset, achieved over 90% accuracy, specifically 90.02%, and reported a strong validation accuracy of 85.33% [24]. A vision-based method involving a Firebird V Robot and neural networks was employed for precise disease detection [25]. Research explored various segmentation techniques that enhance crop disease detection capabilities. Methods such as region-based segmentation, partition clustering, and edge detection are used to categorize images for disease identification [26]. Focusing on plant disease diagnosis via machine learning, the article examined different methods concerning their accuracy in crop disease detection. CNNs proved effective due to their feature extraction capabilities, high accuracy, and suitability for handling large datasets. Collaboration among these CNN systems enables automated plant disease detection, removing the need for human observation in plant health inspections and increasing the precision of disease management measures [27].

This study aims to detect rice diseases using a CNN-based model and Naïve Bayes algorithm, exploring Plant Disease Detection Using IoT devices for real-time monitoring of plant conditions [28]. The proposed methodology advocates for the reliability of the Radial Basis Function Neural Network (RBFNN) in predicting diseases, especially in rice plants. The research team conducted an organized assessment of different image processing procedures used in plant disease detection, focusing on major approaches to enhance image quality and classification accuracy. The researchers applied noise filtering to improve image clarity by removing disturbances and used contrast enhancement to boost image brightness for better detection of disease areas. The study investigated the use of Support Vector Machines to identify diseases in high-dimensional spaces alongside k-nearest Neighbors for classifying images by comparing them to similar nearby points, aiming for more accurate disease diagnosis [29].

The research team behind the project developed disease recognition and blast classification using deep Convolutional Neural Networks (CNN). By analyzing a diverse set of rice leaf images, this system accurately identified healthy and diseased conditions. This diagnostic technology achieved high accuracy by optimizing tumor architecture within deep learning networks and refining network parameters, demonstrating its potential for real-time agricultural disease detection systems [30]. The article explores various methods for feature extraction, image segmentation, and plant disease classification through image processing. It presents three segmentation techniques: boundary detection, spot detection, and the use of k-means clustering and Otsu thresholding to isolate affected areas. Two feature extraction methods include color co-occurrence analysis and leaf color separation based on H and B components. The research employs ANN, BPNN, modified SOM, and multiclass SVM to improve the validity of disease detection in classification tasks [31].

The RiceTalk AI model is an IoT device that reduces the administrative costs associated with real-time forecasts. RiceTalk achieves an 89.4% prediction accuracy for rice blast detection in its current form 7. Vision-based detection of plant leaf diseases using color segmentation with the Firebird V Robot was conducted, utilizing high-quality camera images and neural networks to identify affected leaf areas accurately. The potential for developing an innovative, efficient, and rapid plant disease recognition system is highlighted, and a systematic review discusses crop disease detection through image processing techniques. Various image preprocessing methods are outlined, such as noise filtering and contrast enhancement [25]. In delineating classification and segmentation techniques for plant disease recognition, region-based segmentation and neural network-based classification methods are emphasized [26]. Research reviews machine learning techniques for diagnosing plant diseases, noting the effectiveness of CNN in image classification tasks [27]. The article stresses the importance of early and accurate disease detection in protecting crop yields and the national economy, using image processing techniques and the RBFNN classifier for rice plant disease classification [32]. Research articles confirm the reliability of RBFNN in disease prediction and maintain a comprehensive database for training and testing purposes [29].

The system employs a deep CNN model for recognizing rice blast disease, creating a dataset of images from infected and healthy rice leaves, and demonstrating the effectiveness of CNN in real-world disease detection. They develop a comprehensive dataset for rice blast disease detection, which benefits future research efforts. Additionally, they compare the performance of CNN with Softmax and CNN with SVM, finding both to be equally effective [33]. The decision tree algorithm achieved over 97% accuracy over 97% in detecting rice diseases, based on the test dataset, ensuring reliable classification results. The evaluation was conducted using a strict 10-fold cross-validation approach, which assessed the model’s ability to generalize to different parts of the dataset. The results confirm the decision tree algorithm’s effectiveness in classifying the three rice diseases [34].

The research explores the importance of features in deep learning applications for image detection tasks, including rice leaf disease identification. It introduces a collection of 5,932 pictures which include bacterial blight, blast, brown spot, and tungro diseases. The study evaluates models using transfer learning and deep feature extraction combined with SVM for analysis. SVM performance improves when integrated with deep features, with ResNet50 + SVM achieving an F1-score of 0.9838 for top results. The first fully connected layers within VGG16, VGG19, and AlexNet classify images more effectively than deeper network layers. CNN models outperform traditional methods like LBP, HOG, and GLCM with SVM in the evaluation [35].

In summary, the literature highlights the importance of automated plant disease detection using image processing. Techniques like segmentation, feature extraction, and classification algorithms help accurately identify and classify diseases. Incorporating IoT devices and employing deep learning models, especially CNN, shows promising results in the field. Table 1 outlines related work on contour-based classification for accurately diagnosing rice leaf diseases.

**Table 1:** Related work of contour-based classification for accurate diagnosis of rice leaf diseases.

Ref.	Dataset	Aim of Study	Methodology	Results	Limitations
[0.5ex] [36]	Rice Oryza sativa	To classify automatically rice leaf diseases	InceptionV3	Accuracy: 90.77%	Need accuracy improvement
[37]	Rice leaf disease	Detection of rice leaf diseases	DenseNet201	Accuracy is 100%	Model faces overfitting as loss must be zero in this case when accuracy becomes 100%
[38]	Rice leaf disease	To classify diseases using pre-trained deep learning models	VGG16, InceptionV3 and ResNet152V2	Models achieve accuracy and F1-score with 100%	Models are likely overfitting
[39]	Rice classification	The study focuses on early leaf disease detection	CNNs, KNNs classifier	Accuracy is 99%	Accuracy may improve
[40]	Paddy rice leaf	To classify bacterial and fungal diseases	EfficientNet-b0	97.74% accuracy	Limited categorization, restricted generalizability
[41]	Rice disease	Detect rice leaf using an UAV-based UAV system	Orange 3.26	86.7% Accuracy	Limited dataset, framework is not enough to continue monitoring crop diseases
[42]	Rice disease	Focus on the classification of dice leaf diseases	Image Processing, ML Algorithms	MDC model: 89.23% accuracy and KNN model: 87.02% accuracy	Limited dataset, i.e., 115 images only used for model training
[43]	Rice leaf disease	Rice leaf disease classification using CV	Computer Vision Algorithms (Texture, Color Descriptor), SVM LBP HOG and SVM	SVM achieves 92.5% accuracy and ANN achieves 87.5% accuracy	High-dimensional feature vector requires reduction
[44]	Rice leaf disease	Study focuses on extracting features from an image dataset, then uses an ML algorithm	LBP, HOG and SVM	94.6% accuracy achieved using SVM	Further research is needed to improve the accuracy
[45]	Rice leaf disease	Classification of rice disease using an image processing method	Image Processing Feature Extraction Technique, ECB Classifier, Morphological Method	Accuracy achieves 86.58% and F1-score 87%	The poor accuracy is a model
[46]	Rice leaf disease	Research focuses on the detection of rice leaf disease	Morphological Method, ML Algos (Bayes’ & SVM)	Bayes’ model: 79.5% accuracy and SVM: 68.1% accuracy	Low accuracy rate and a lack of methodology detail
[47]	Rice leaf disease	Detection of rice leaf disease using DL	VGG-19 TL model	Average accuracy achieves 96.08%	Challenging implementation of the system in ample agricultural land
[1]	Rice leaf disease	To classify rice leaf diseases with a lightweight architecture	SqueezeNet	99.30% accuracy	Evaluation limited to classification task only
[2]	Rice leaf disease	To classify rice leaf diseases using deep learning	Hybrid CNN-VGG Deep Learning model	Accuracy: 95.60%	Lacks detailed evaluation metrics like precision and recall
[13]	Rice leaf disease	To classify rice leaf diseases using transfer learning	VGG-19 with Grad-CAM	Accuracy: 95.88%, F1-score: 0.9530	Limited generalization and less accuracy

## Proposed methodology

The proposed methodology employs pre-trained deep learning models, including InceptionV3, DenseNet201,ResNet152V2,EfficientNetV2L and MobileNetV2 to support the detection of rice leaf diseases through the GrabCut segmentation and contour detection processes, which enhance result accuracy. These images are initially used to train the deep learning architectures, extracting high-level features from rice leaf images via transfer learning. The model’s architecture is fine-tuned to better differentiate among common diseases such as brown spots, leaf blasts, and leaf blight. Contour detection is also applied to refine the identified disease regions, enabling precise lesion localization on leaf surfaces after classification. Extensive experimental results demonstrate the effectiveness of these methods, surpassing traditional approaches in both accuracy and computational speed. Ultimately, this approach provides farmers with prompt and accurate information for crop management.

### Proposed solution framework

**Fig 4:**
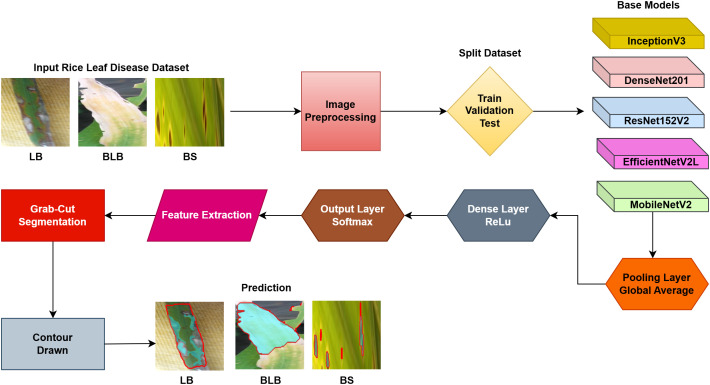
Proposed solution framework of Enhanced Rice Leaf Disease Classification via Contour-Driven Segmentation and Optimized Deep Transfer Learning Architectures.

The following Fig. 4 illustrates a workflow model showing how deep learning models are implemented for rice leaf disease classification. The architecture of the CNN approach used for classifying plant images infected with diseases (for BLB, BS, and LB) processes and feeds a dataset of preprocessed leaves. Next, the data is split into three separate datasets (train, validation, and test), on which three base models are trained using InceptionV3, DenseNet201,ResNet152V2, EfficientNetV2L and MobileNetV2. The features pass through a dense ReLU layer after a global average pooling layer that refines them. A softmax layer performs the final classification. The GrabCut segmentation process extracts diseased areas, enabling subsequent contour drawing to improve visualization quality. The segmentation of images, along with their classification, allows technicians to locate disease areas very accurately. This method provides both precise identification and real-time automated detection of diseases through its system.

The images from the input dataset used to evaluate performance are diseased leaves, including Bacterial Leaf Blight (BLB), Brown Spot (BS), and Leaf Blast (LB), as commonly accepted in the industry. We then proceed to image processing, followed by splitting the dataset into training, validation, and testing sets before training various base models (InceptionV3, DenseNet201, ResNet152V2, EfficientNetV2L and MobileNetV2) for feature extraction. These models are connected to additional layers: a global average pooling layer and a dense layer with ReLU activation, topped with the final softmax output layer. This setup allows the model to predict leaf diseases given an input dataset. In order to make the proposed approach transparent and reproducible, the details of the whole implementation have been publicly available on a separate GitHub repository. This research has the source code, model configurations, and structured arrangement of the dataset organized in this research in order to enable validation and additional studies. The respective reference [48] has been utilized to give the relevant recognition and allow direct access to the experimental framework.

The research project uses a high-speed transfer learning method based on InceptionV3, DenseNet201, ResNet152V2, EfficientNetV2L and MobileNetV2 models to classify rice leaf diseases. It focuses on quick training with minimal preprocessing, utilizing a stable learning rate on GPUs, and monitors performance and time epoch by epoch.

- **Environment setup**: Load necessary libraries (NumPy, TensorFlow, Matplotlib) and set paths to dataset directories.
- **Data preparation**: Basic augmentation on images using ‘ImageDataGenerator‘, producing batches of train/validation data with a batch size of 32.
- **Model architecture**: Load the frozen (InceptionV3, DenseNet201, ResNet152V2, EfficientNetV2L and MobileNetV2) pre-trained models, add a global average pooling layer and a softmax-optimized classification head.
- **Training configuration**:
  – Optimizer: SGD (learning rate = 0.001, momentum)
  – Batch size: 32
  – Hardware: T4x2 GPU configuration
  – Callbacks: Model checkpoint and epoch-time logging
- **Training**: Run for 30 epochs with fewer steps per epoch, record timing data, and plot epoch time. The stable batch size ensures parallel efficiency.

Stochastic gradient descent (SGD) at a learning rate of 0.001 and a momentum of 0.9 are used to optimize the model, which can be easily and efficiently converged during training. The batch size used is 32 and the number of epochs is 30, with a constant random seed of 42 so that the results of the experiment can be reproduced and have a consistent result as shown in Table 2.

**Table 2:** Training hyperparameters and configuration.

Parameter	Value
Optimizer	SGD
Learning Rate	0.001
Momentum	0.9
Batch Size	32
Epochs	30
Seed	42

- **Results analysis:**
  – **Training Performance**: Report total/average training time, final accuracy, and validation performance. Note stable batch processing (size 32) and potential instability from the significant learning rate.
  – **GrabCut Segmentation**: Disease regions were isolated and highlighted in red, using adaptive foreground-background separation.
  – **Contour Detection**: Effected disease boundaries were delineated and marked in blue via morphological post-processing.

#### Reproducibility Details.

The proposed framework can be reproduced because the information about the dataset, preprocessing, model setup, and training is provided in detail. The models were all done with the TensorFlow deep learning framework and they were trained on an NVIDIA T4x2 environment. They split the dataset into training, validation and test subsets, image materials were resized and normalized, and then it was trained. Hyperparameters such as learning rate 0.001, SGD optimizer setup, batch size = 32, and epochs = 30 and each model was trained four times using different random seeds (42, 100, 202, 512), so that they can be fairly compared. Accuracy, precision, recall, F1-score, mean IoU, and mean Dice were used to evaluate it.

### Model architecture comparison

InceptionV3 was very efficient in terms of accuracy and exhibited good behavior during the entire training process. As shown in Table 3 InceptionV3, balanced design with 23.8M parameters and 2048-dimensional feature space can well represent discriminative features at 299x299, input resolution. The model was able to keep the performance steady during the training and there were no indications of overfitting or instability.

**Table 3:** Model Architecture Comparison.

Model	Input Size	Feature Dimension	Parameters
InceptionV3 [49]	299×299	2048	23.8M
DenseNet201 [50]	224×224	1920	20M
ResNet152V2 [51]	224×224	2048	60M
EfficientNetV2L [52]	480×480	1280	118M
MobileNetV2 [53]	224×224	1280	3.4M

### Dataset acquisition

The dataset used in the current study has been posted and made publicly available on Zenodo, ensuring transparency and reproducibility of the research. The DOI can be cited to reference the data source [54]. Data augmentation was applied to expand the dataset. The images in the collection are categorized into three groups: brown spot, bacterial blight, and leaf blast. Initially stored as RGB images, these inputs can be converted into any format you prefer.

### Dataset image resolution distribution

Images in the Rice Leaf Disease Dataset have an uneven spatial resolution with a range of width pixels of 286−3081(mean=501.8) in width and the range of height pixels of 88−900(mean=337.7).

**Fig 5:**
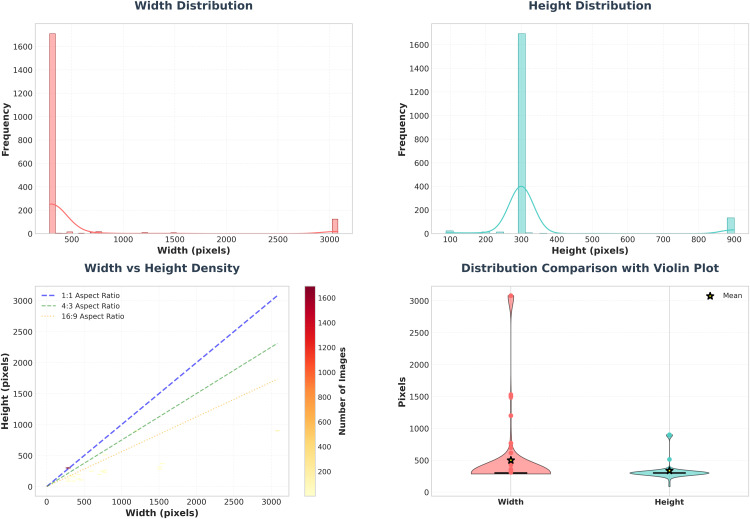
Dataset image resolution distribution.

According to the statistics of image sizes, the dataset consists of images of size 286–3081 pixels in width and 88–900 in height, with an average size of about 502x338, and the low 25 th −75 th percentiles (all equal to 300 pixels) meaning that most images are evenly small (around 300x300), with a few very large images that result in high standard deviations and skewart the mean, at least in width, as shown in Fig 5. This variation in dimensions provides a representative sampling of different capture conditions in agricultural imaging applications. The resolution range of the dataset supports stable model training, thanks to the introduction of consistent features with scale invariance across different levels of magnification.

### Image pre-processing

Under Image Pre-Processing, data augmentation is a key concept in machine learning, (especially for image-related tasks. During training, the dataset is used to train the model. However, sometimes using only the central data is not enough, so data augmentation techniques are helpful. Performing data augmentation involves applying various random transformations such as rotating, flipping (horizontal/vertical), zooming in and out, and shearing; these operations help prevent the model from overfitting by reducing reliance on specific parts of the image.

Data augmentation was applied exclusively to the training dataset. The validation and test datasets remained unchanged to ensure unbiased performance evaluation and prevent data leakage.

#### Dataset before augmentation.

The rice leaf disease dataset contains 354 image files. The training set has 246 images, including 85 Bacterial Leaf Blight (BLB), 98 Brown Spot (BS), and 63 Leaf Blast (LB). The validation set includes 70 images, divided into 24 (BLB), 28 (BS), and 18 (LB). The test set has 38 images: (i.e., 13 (BLB), 15 (BS), and 10 (LB) extitciter1. This distribution shows a class imbalance, but it is moderate, considering the training set, where BS has more samples than LB. This imbalance can affect model performance and may require augmentation or resampling to achieve balanced learning. The dataset’s size is 30.99 MB.

#### Dataset after augmentation.

Table 4 shows details; the balanced and augmented rice leaf disease dataset has an increased number of training images, 1,338 in total, 446 each for Brown Spot, Leaf Blast, and Bacterial Leaf Blight, making the class distribution perfectly balanced. The validation and test sets are also proportioned (128: 64 images per class, respectively), ensuring a valid evaluation. Such homogeneity removes any bias and enhances the generalizability of models, as well as the accuracy of disease classification across all categories. The overall total dataset samples images are 1914 [2].

**Table 4:** Image counts per class.

Classes	Train	Validation	Test
Brown Spot	446	128	64
Leaf Blast	446	128	64
Bacterial Leaf Blight	446	128	64

Data augmentation is one of the most essential steps to enhance our deep learning model’s performance, especially when training with limited images. This is what Augmentor does to make datasets less uniform compared to how they appear in the real world. We used the Augmentor Python library. It is very flexible for performing these augmentations. It allows you to create pipelines that apply transformations sequentially or probabilistically. It is an efficient library for image classification and can customize data augmentation based on the nature of the dataset.

Let the input image be denoted as $I\in {\mathbb{R}}^{H\times W\times C}$, where *H* is the height, *W* is the width, and *C* is the number of color channels (typically *C* = 3 for RGB images).

#### Normalization.

To standardize the intensity distribution, we normalize the image using the dataset’s mean μ and standard deviation σ:


$$I_{\text{norm}}=\frac{I-\mu }{\sigma }$$

#### Resizing.

Normalized images are resized to match the input requirements of the deep models used in our framework:


$$I_{\text{resized}}=\text{Resize}(I_{\text{norm}},(H^{\prime },W^{\prime }))$$

Where $\left(H^{\prime },W^{\prime }\right)$ is defined as:


$$(H^{\prime },W^{\prime })=\left\{ \begin{array}{cc} 299\times 299, & \text{for InceptionV3}\\ 224\times 224, & \text{for DenseNet201 and ResNet152V2} \end{array}\right.$$

### Dataset splitting

The dataset must be split for machine learning or deep learning models because it helps them become more generalized and applicable to unseen data. You already know a common way to divide your dataset into training, validation, and test sets because each serves a slightly different purpose in model building. Alternatively, this can be done using a Python library called splitfolders, where you select the ratio and ensure that the class distribution is preserved.

Let, D be the entire dataset consisting of N images. It is split into three sets:


$$\begin{array}{cc} D & =D_{\text{train}}\cup D_{\text{val}}\cup D_{\text{test}}\\ \text{with}\; & |D_{\text{train}}|=0.7N,\;|D_{\text{val}}|=0.2N,\;|D_{\text{test}}|=0.1N \end{array}$$

Every part of the dataset shows examples from the three categories.


$$\begin{array}{cc} D_{\text{train}} & =\overset{\text{BLB}}{\underset{\text{train}}{D}}\cup \overset{\text{BS}}{\underset{\text{train}}{D}}\cup \overset{\text{LB}}{\underset{\text{train}}{D}}\\ D_{\text{val}} & =\overset{\text{BLB}}{\underset{\text{val}}{D}}\cup \overset{\text{BS}}{\underset{\text{val}}{D}}\cup \overset{\text{LB}}{\underset{\text{val}}{D}}\\ D_{\text{test}} & =\overset{\text{BLB}}{\underset{\text{test}}{D}}\cup \overset{\text{BS}}{\underset{\text{test}}{D}}\cup \overset{\text{LB}}{\underset{\text{test}}{D}} \end{array}$$

Table 4 shows the classes of the rice leaf diseases dataset with the image counts per class for train, validation, and a test directory.

The overall image dataset, which includes three classes Brown spot, Leaf blast, and Bacterial leaf Blight consisting of training, validation, and test sets, has a range of 1914 files and has a total size of 465 MB. All classes are balanced, as balancing the dataset is considered best practice for accurately evaluating performance metrics. Table 4 presents three categories: Brown Spot, Leaf Blast, and Bacterial Leaf Blight, as train set 446 images in the training set, 128 images and test set 64 images of each corresponding classet. It also provides precision and recall for a balanced split, which are valuable metrics to evaluate the model’s performance and prevent class imbalance issues.

The dataset was first split into training (70%), validation (20%), and testing (10%) using stratified sampling. Data augmentation was then applied exclusively to the training subset to prevent information leakage. Random seed was fixed at 42 for reproducibility.

**Pseudo-procedure:**

1. Load original dataset
2. Stratified split (70/20/10)
3. Apply augmentation only to *D*_train_
4. Keep *D*_val_ and *D*_test_ unchanged

### Mathematical model for the proposed framework

#### Feature extraction.

The obtained feature vectors are in the format (X, Y), where ’X’ indicates the number of images from the dataset and ’Y’ represents the feature dimension determined by the selected model. The dataset contains a total of 1914 images, while the feature dimension varies depending on the model used: it is 2048 features for InceptionV3 and ResNet152V2, and 1920 features for DenseNet201.

Pre-trained CNNs (InceptionV3, DenseNet201, ResNet152V2, EfficientNetV2L and MobileNetV2) are used to extract deep features from segmented input images. Let *x* represent the preprocessed and segmented input image. The deep feature representation 𝐟 is obtained as:


$$𝐟={\text{CNN}}_{\theta }(x)\in {\mathbb{R}}^{H\times W\times C}$$

Where:

- ${\text{CNN}}_{\theta }$ denotes a pre-trained convolutional neural network with parameters θ (InceptionV3, DenseNet201, ResNet152V2, EfficientNetV2L and MobileNetV2).
- *x* is the input image after normalization, resizing, and segmentation.
- 𝐟 is the output feature map.
- *H*, *W*, *C* are the height, width, and number of channels in the feature map, respectively.

We obtain these features after applying the Global Average Pooling (GAP) method to the final convolutional layer. The resulting elements are then used for subsequent processing steps, including classification and analysis.

#### Global average pooling layer.

Global Average Pooling (GAP) is a specialized pooling layer in Convolutional Neural Networks (CNNs) that replaces fully connected (FC) layers and offers several benefits. It calculates the mean of the spatial values in each feature map, resulting in one value per channel. This effectively converts the feature maps into a fixed-size output regardless of the input image size and eliminates the need for FC layers.

The Global Average Pooling layer reduces each feature map to a single value by averaging over all spatial dimensions. Given an input feature map $Fin{\mathbb{R}}^{H\times W\times D}$, where *H* is height, *W* is width, and *D* is depth (number of channels), the operation is defined as:


$$\text{GAP}(F{)}_{d}=\frac{1}{H\times W}\sum_{i=1}^{H}\sum_{j=1}^{W}F_{i,j,d}$$

where:

- $\text{GAP}(F{)}_{d}$ is the output for channel *d*
- *F*_*i*,*j*,*d*_ is the activation at position (*i*,*j*) in channel *d*
- *H* × *W* represents the total spatial area being averaged

#### Dense layer with ReLU activation function.

A dense layer with ReLU (Rectified Linear Unit) activation function is a fundamental component of many networks. It is essentially a layer of neurons where each neuron connects to all neurons in the previous layer (hence “dense”), and the ReLU activation function introduces non-linearity. ReLU outputs zero for negative input values and passes positive input values unchanged, helping the network learn complex patterns.

The dense layer inputs features into a lower-dimensional space, followed by ReLU activation:


$$h=\text{ReLU}(W_{g}\cdot g+b_{g})$$

where:

- $g\in {\mathbb{R}}^{n}$ is the input feature vector (typically from a pooling layer)
- $W_{g}\in {\mathbb{R}}^{m\times n}$ is the learnable weight matrix
- $b_{g}\in {\mathbb{R}}^{m}$ is the bias vector
- ReLU(x)=max(0,x) is the rectified linear unit activation function
- $h\in {\mathbb{R}}^{m}$ is the output feature vector

#### Softmax Output Layer.

The softmax function is a type of activation function that is used for multi-classification tasks.

Let the output logits from the final dense layer be represented as:


$$𝐳=[z_{1},z_{2},z_{3}]$$

For our rice leaf disease classification task, we consider three categories:

- *C*_1_: Bacterial Leaf Blight (BLB)
- *C*_2_: Brown Spot (BS)
- *C*_3_: Leaf Blast (LB)

The Softmax function computes the probability for each class *C*_*i*_ as:


$$P(C_{i}|𝐳)=\frac{e^{z_{i}}}{\sum_{j=1}^{3}e^{z_{j}}},\;\text{for }i=1,2,3$$

where:

- *z*_*i*_ is the logit score for class *C*_*i*_
- $\sum_{j=1}^{3}e^{z_{j}}$ serves as the normalization factor (ensuring $\sum_{i=1}^{3}P(C_{i}|𝐳)=1$)
- $P(C_{i}|𝐳)\in [0,1]$ represents the model’s predicted probability for class *C*_*i*_

#### GrabCut segmentation.

The GrabCut algorithm segments an image by minimizing an energy function *E* that separates foreground (e.g., diseased regions) from the background. The energy function is defined as:


$$E(\alpha ,k,\theta ,z)=\sum_{n}D(\alpha_{n},k_{n},\theta ,z_{n})+\lambda \sum_{(m,n)\in 𝒩}V(\alpha_{m},\alpha_{n},z)$$

**Components of the energy function:**

- **Data Term (Unary Potential)**: Penalizes mismatches between pixel intensities and the Gaussian Mixture Model (GMM) for foreground/background regions.
- **Smoothness Term (Pairwise Potential)**: Encourages neighboring pixels with similar intensities to share the same label.

**Data term:**


$$D(\alpha_{n},k_{n},\theta ,z_{n})=-\log\left(\text{GMM}(z_{n}|\alpha_{n},k_{n},\theta )\right)$$

**Parameters:**

- $\alpha_{n}\in \{0,1\}$: Binary label for pixel *n* (0 = background, 1 = foreground).
- *k*_*n*_: Index of the GMM component assigned to pixel *n*.
- θ: Parameters of the GMM (mean, covariance).
- *z*_*n*_: Intensity value of pixel *n*.

**Smoothness term:**


$$V(\alpha_{m},\alpha_{n},z)=\delta (\alpha_{m}\neq \alpha_{n})\cdot \exp\left(-\beta \|z_{m}-z_{n}{\|}^{2}\right)$$

**Parameters:**

- λ: Weight balancing the influence of the smoothness term.
- 𝒩: Set of neighboring pixel pairs.
- β: Sensitivity to intensity contrasts ($\beta ={\left(2\langle \|z_{m}-z_{n}{\|}^{2}\rangle \right)}^{-1}$).
- δ(·): Kronecker delta function (1 if labels differ, zero otherwise).

What makes the GrabCut method new is the combination with deep CNN features, where low-level, pixel-level cues are determined by high-level semantic features produced by InceptionV3, DenseNet201 ResNet152V2, EfficientNetV2L and MobileNetV2. With this integration, more precise boundary refinement and strong foreground extraction are achieved, especially in complicated agricultural images.

#### Contour detection.

Contouring is a fundamental technique in computer vision and image processing, with boundary detection used to identify the edges of an object in an image. OpenCV, a robust open-source computer vision library, provides the cv2.findContours() function to obtain contours in an image and detect the contour. In this tutorial, we describe the basic concepts, contouring steps, retrieval modes, and approximation methods available in OpenCV to understand better contouring.

After segmenting the image, gradient computation is performed to detect contours. The gradient magnitude *G*(*x*,*y*) at each pixel (*x*,*y*) is calculated as:


$$G(x,y)=\sqrt{{\left(\frac{\partial I}{\partial x}\right)}^{2}+{\left(\frac{\partial I}{\partial y}\right)}^{2}}$$

where:

- *G*(*x*,*y*) represents the gradient magnitude at pixel location (*x*,*y*)
- $\frac{\partial I}{\partial x}$ and $\frac{\partial I}{\partial y}$ are the horizontal and vertical image derivatives, respectively

**Edge detection:**

The system employs either Canny or Sobel edge detection filters to identify prominent edges. These edges are then used to construct bounding boxes around regions of interest.

Key features of this process:

- **Canny Edge Detector**: Provides optimal edge detection with noise suppression
- **Sobel Operator**: Efficiently approximates gradient magnitudes
- **Bounding Box Generation**: Selected edges are connected to form rectangular regions enclosing detected features

### Pre-trained transfer learning models

In image classification tasks, popular Convolutional Neural Networks (CNNs) are InceptionV3, DenseNet201, ResNet152V2, EfficientNetV2L and MobileNetV2.

#### InceptionV3 architecture.

The architecture of InceptionV3 is a deep 48-layer model, with features extracted from over a million randomly selected images from the ImageNet database. It can identify image categories, and the input size is 299 × 299 pixels. The InceptionV3 transfer learning model demonstrates both high accuracy and efficient feature inspection when detecting rice leaf diseases. The training process becomes more efficient by using pre-trained weight parameters to distinguish disease areas from background noise during detection. Its optimized framework enhances generalization, allowing the model to detect diseases even when datasets are limited.


$$\begin{array}{cc} Y & =\text{Concat}[{\text{Conv}}_{1\times 1}(x),\;{\text{Conv}}_{3\times 3}({\text{Conv}}_{1\times 1}(x)),\\ & \;{\text{Conv}}_{3\times 1}({\text{Conv}}_{1\times 3}({\text{Conv}}_{1\times 1}(x))),\;{\text{AvgPool}}_{3\times 3}(x)] \end{array}$$ (1)

Equation 2 describes a structure inspired by the InceptionV3 model, where multiple convolutional and pooling components operate simultaneously. The input x undergoes sequential processing through three different convolution operations: 1*x*1, 3*x*3 and asymmetric convolution, followed by an average pooling operation of 3*x*3. The depth-wise combination of all feature maps produces the output Y.

The convolution operation in InceptionV3 is represented as:


$$y_{i,j,k}=\sum_{m,n}x_{i+m,j+n}\cdot w_{m,n,k}$$

where *x* is the input feature map, *w* is the convolutional kernel, and *y* is the output feature map.

**Inception module A branches:**

- 1x1 Convolution
- 5x5 Convolution
- 3x3 Convolution followed by another 3x3 Convolution
- 3x3 Max Pooling followed by 1x1 Convolution

**Inception module B branches:**

- 1x1 Convolution
- 1x7 Convolution followed by 7x1 Convolution
- 1x7 Convolution followed by 7x1 Convolution followed by another 1x7 Convolution and 7x1 Convolution
- 3x3 Max Pooling followed by 1x1 Convolution

**Inception module C branches:**

- 1x1 Convolution
- 1x3 Convolution followed by 3x1 Convolution
- 1x3 Convolution followed by 3x1 Convolution followed by another 1x3 Convolution and 3x1 Convolution
- 3x3 Max Pooling followed by 1x1 Convolution

The auxiliary classifier is represented as:


$$\hat{y}=\text{Softmax}(W\cdot \text{GlobalAvgPool}(x)+b)$$

where *x* is the input feature map, *W* and *b* are the weights and biases, and $\hat{y}$ is the predicted output.

The final classification layer uses:


$$\hat{y}=\text{Softmax}(W\cdot \text{GlobalAvgPool}(x)+b)$$

where *x* is the input feature map, *W* and *b* are the weights and biases, and $\hat{y}$ is the predicted output.

**Fig 6:**
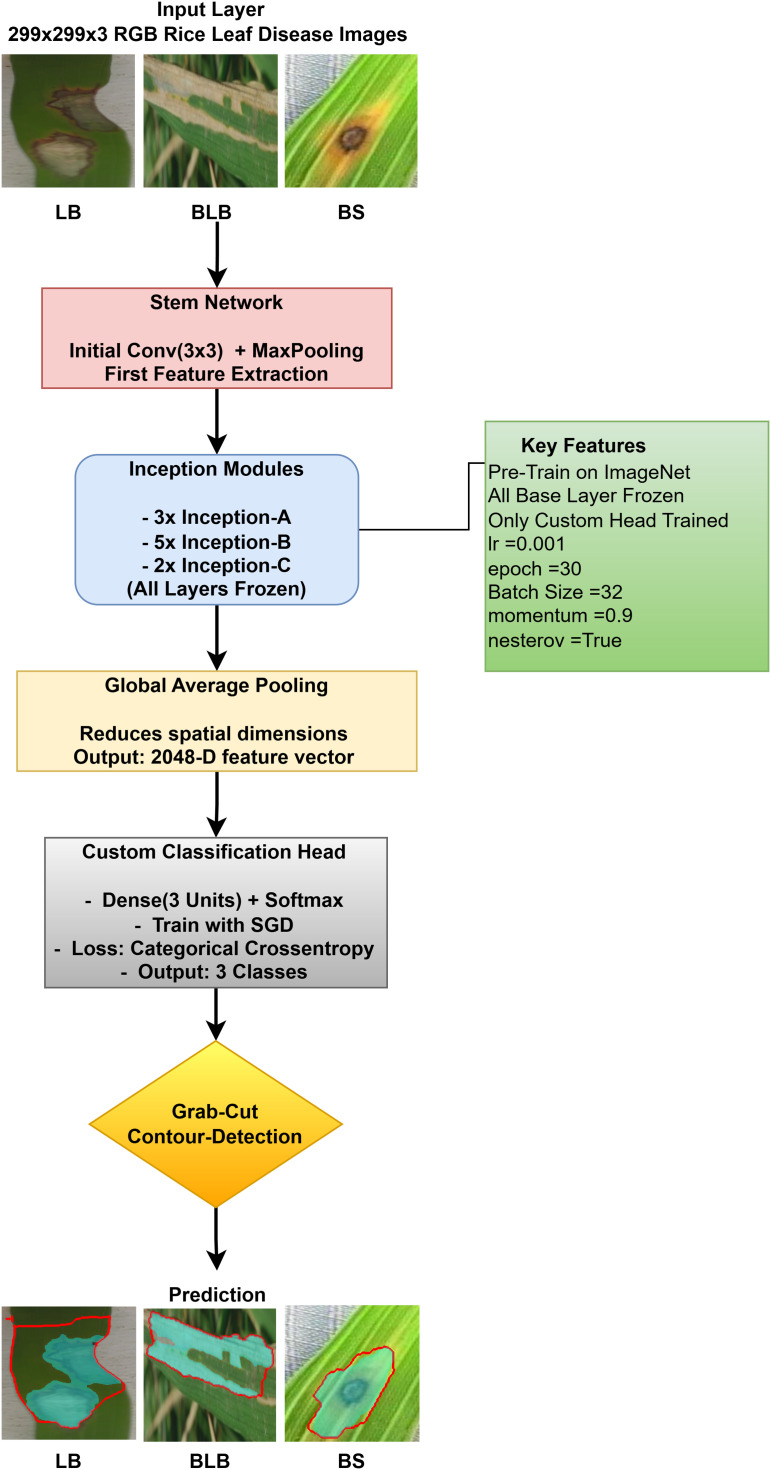
InceptionV3 architecture with proposed solution.

The proposed solution combines the use of InceptionV3 with contour detection to extract features during rice leaf disease classification. This Fig. 6 illustrates InceptionV3, which is essentially an architectural framework that functions as one of the more advanced optimized convolutional neural network (CNN) designs for extracting features from an image.

#### Mathematical model for the InceptionV3 architecture with proposed solution.

**Image representation for InceptionV3**

Let $I\in {\mathbb{R}}^{H\times W\times C}$ be an input RGB image of size *H* × *W* and *C* = 3 (color channels). This line defines a 3D tensor I representing an RGB image with height H, width W and 3 color channels. It’s commonly used in image processing and deep learning contexts.

**Global average pooling layer**

The Global Average Pooling (GAP) layer reduces each feature map to a single value by taking the average of all spatial dimensions (height and width). Given an input tensor $𝐗\in {\mathbb{R}}^{H\times W\times C}$, where *H* is the height, *W* is the width, and *C* is the number of channels, the output $𝐲\in {\mathbb{R}}^{C}$ is computed as:


$$y_{c}=\frac{1}{H\times W}\sum_{i=1}^{H}\sum_{j=1}^{W}x_{i,j,c}$$

where:

- *y*_*c*_ is the output for the *c* -th channel,
- *x*_*i*,*j*,*c*_ is the value at position (*i*,*j*) in the *c* -th channel of the input 𝐗.

**Dense Layer with ReLu**


$$h=\text{ReLU}(W_{1}X+b_{1})$$

- $W_{1},b_{1}:$ weights and bias for the dense layer
- ReLU: ReLU(x)=max(0,x)

The successfully processed extracted features pass to a dense ReLU layer where they undergo deeper investigation.

**Output Layer with Softmax for Classification**

Assume *K* classes (e.g., BLB, BS, LB):


$${\hat{y}}_{i}=\frac{\exp(W_{2}^{\left(i\right)}h+b_{2}^{\left(i\right)})}{\sum_{j=1}^{K}\exp(W_{2}^{\left(j\right)}h+b_{2}^{\left(j\right)})}$$

- ${\hat{y}}_{i}$: probability of class *i*
- $W_{2}^{\left(i\right)},b_{2}^{\left(i\right)}$: weights and biases of output layer

A softmax output layer determines the disease categories in the final classification stage. This method provides precise identification of infected areas through localized control of diagnosis. It delivers accurate results for detecting infected zones by using localized testing procedures. Targeted and reliable disease analysis is achieved through careful regional monitoring of diagnostic processes, which helps minimize errors.

**Loss function (cross-entropy)**


$$ℒ=-\sum_{i=1}^{K}y_{i}\log({\hat{y}}_{i})$$

- *y*_*i*_: true label (one-hot encoded)
- ${\hat{y}}_{i}$: predicted probability Cross Entropy is type of loss function use for classification purpose.

**Optimization**

Backpropagation with SGD optimizer is used to minimize ℒ:


$$\theta \leftarrow \theta -\eta \cdot \nabla_{\theta }ℒ$$

- η: learning rate
- θ: network parameters

**Feature Extraction using CNN**

Let $f_{\theta }(I)$ represent the deep CNN (e.g., InceptionV3) parameterized by weights θ. The CNN extracts feature maps:


$$X=f_{\theta }(I)$$

where $X\in {\mathbb{R}}^{d}$ is the high-dimensional feature vector after global average pooling.

**GrabCut Segmentation for InceptionV3**

The GrabCut algorithm is based on graph cuts and models the image as a Markov Random Field (MRF). It iteratively estimates the foreground (disease region) *F* and background *B*:


E(L,θ,z)=U(L,θ,z)+V(L,z)

- *L*: binary label assignment (foreground/background)
- θ: Gaussian Mixture Model (GMM) parameters
- *z*: pixel values
- U(·): data term (fit of pixels to GMMs)
- V(·): smoothness term (penalizes discontinuities)

GrabCut is a segmentation algorithm that models images as a foreground and background, each pixel in an image is modeled as either foreground or background, and the energy functional as shown in above equation [55], is minimized to ensure accurate separation between the foreground and the background.

**Contour detection for InceptionV3**

After segmenting the image, find the gradient lines to detect contours.


$$G(x,y)=\sqrt{{\left(\frac{\partial I}{\partial x}\right)}^{2}+{\left(\frac{\partial I}{\partial y}\right)}^{2}}$$

- G(x,y): Gradient Magnitude at pixel (x,y)

The system detects edges using Canny or Sobel filters and selects the identified edges to create bounding boxes. The InceptionV3 model extracts relevant features that support GrabCut segmentation in identifying areas with diseases. The segmented results then require additional contour detection methods for identifying affected regions.

The approach involves using pre-trained InceptionV3 architecture as shown in Fig 6, where the base layers (Inception modules A/B/C) are kept frozen. The multi-scale features extracted by the architecture are then projected into a global average pooled 2048-D feature. A custom classification head (a dense layer with 3 units + softmax) is trained using SGD to classify three rice disease categories, with a optimized low learning rate (0.001) and trained over 30 epochs. After classification, contour detection and GrabCut are used to localize diseased areas, enhancing interpretability. This method combines transfer learning, efficient fine-tuning, and explainable AI to provide a robust and actionable disease diagnosis in rice plants.

#### DenseNet201 architecture.

The proposed DenseNet201 consists of 201 layers with a unique connection pattern, where each layer is connected to all previous layers, stabilizing feature propagation and reducing the number of parameters. It is trained on ImageNet and supports an input size of 224 *x* 224 pixels. DenseNet201 enhances rice leaf disease detection through dense connectivity that enables efficient feature transfer, reducing information loss. The model design achieves high accuracy with lower computational costs due to its extensive architecture combined with a reduced number of parameters. It provides reliable, precise classification of complex disease patterns thanks to its effective pattern recognition capabilities.


$$\begin{array}{cc} x_{\ell }=\text{Concat}[ & x_{0},x_{1},\ldots ,x_{\ell -1},\\ & {\text{BN-ReLU-Conv}}_{1\times 1}({\text{BN-ReLU-Conv}}_{3\times 3}(x_{\ell -1}))] \end{array}$$ (2)

The expression 2 describes DenseNet201 which enables each network layer to access complete output from all preceding layers. The new feature map calculation includes several sequential operations of BatchNorm followed by ReLU before 3*x*3 Conv and then another BatchNorm, ReLU and 1*x*1 Conv. The resulting output gets concatenated with all past feature maps to create xℓ.

**DenseNet201 architecture**

Input image tensor:


$$x_{0}\in {\mathbb{R}}^{H\times W\times C}$$

Initial processing:


$$x_{1}=\text{MaxPool}({\text{Conv}}_{7\times 7}(x_{0}))$$

Dense Layer operation (per layer *l*):


$$x_{l}=H_{l}([x_{0},x_{1},\ldots ,x_{l-1}])$$

where *H*_*l*_ contains:

- BatchNorm → ReLU → Conv_1×1_ → BatchNorm → ReLU → Conv_3×3_

Transition layer between blocks:


$$x^{\prime }={\text{AvgPool}}_{2\times 2}({\text{Conv}}_{1\times 1}(x))$$

Final classification:


$$\hat{y}=\text{Softmax}(W\cdot \text{GlobalAvgPool}(x)+b)$$

**Fig 7:**
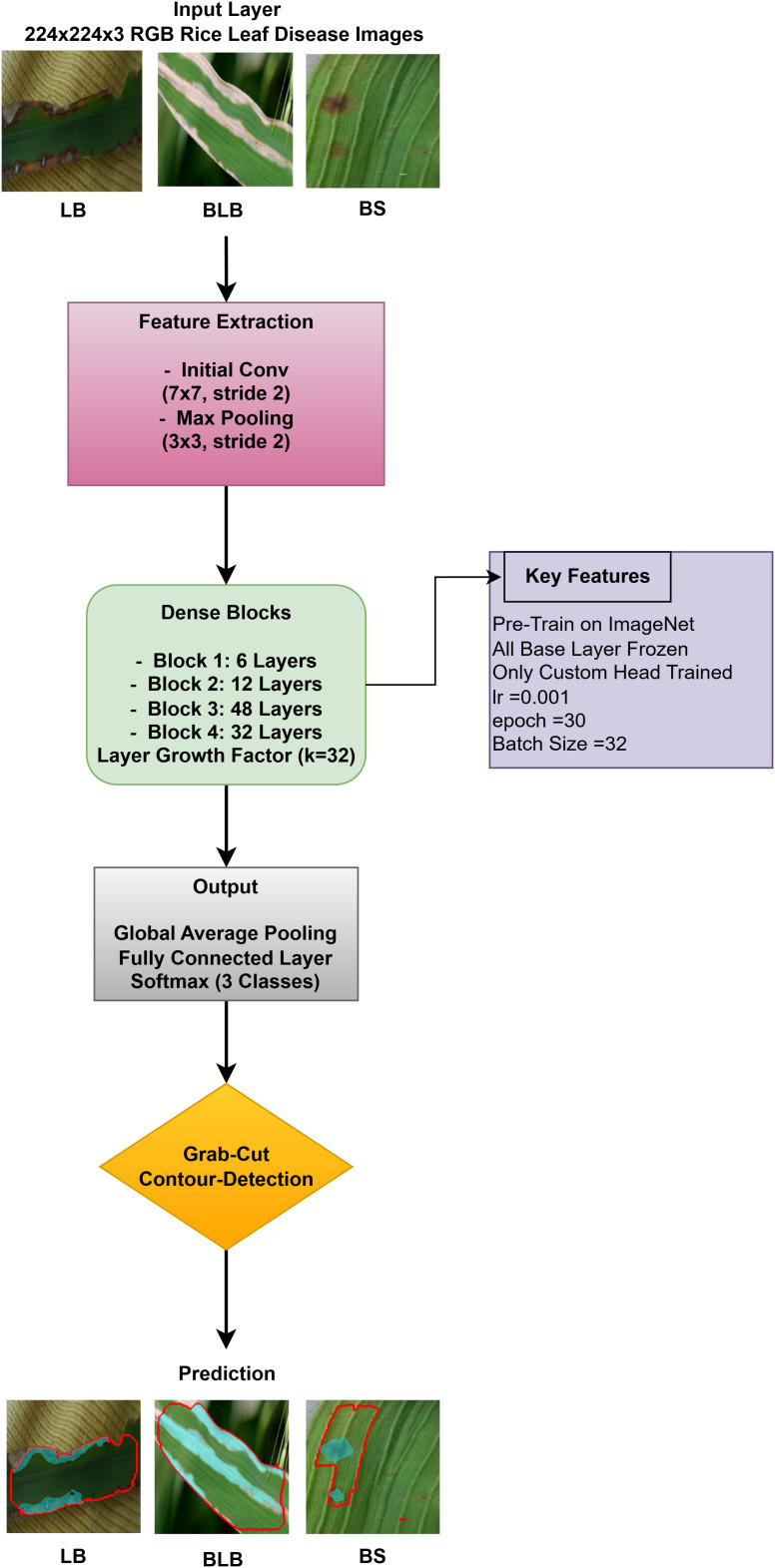
DenseNet201 architecture with proposed solution.

DenseNet201 works in conjunction with contour-based segmentation as an integrated solution for classifying rice leaf diseases. The preprocessing of rice leaf images aims to enhance patterns that specifically indicate the presence of disease. The data distribution comprises three distinct parts used for model assessment. The network employs a global average pooling operation, which reduces feature dimensionality while preserving vital information. The dense ReLU (Rectified Linear Unit) layer serves as a core component to improve the quality of features by enhancing their representation before classification. As an activation function, this layer introduces non-linearity to the model, retaining important feature patterns while eliminating noise, leading to improved accuracy in decision making. The softmax layer generates a probability distribution of target classes immediately after the refinement process to identify the most likely disease category for each image. The output from this step is termed “refracted outputs” because it demonstrates the model’s ability to distinguish among various disease classes through their unique visual patterns. Using DenseNet201, the model extracts deep features via its dense architecture, which optimizes feature propagation and reuse. After feature extraction, the GrabCut segmentation process isolates diseased regions with enhanced accuracy. The detection process then highlights disease-affected areas, enabling more precise analysis of infection patterns, as shown in Fig. 7.

#### Mathematical model for the DenseNet201 architecture with proposed solution.

**Input image representation**

Let:

-
$I\in {\mathbb{R}}^{H\times W\times C}$

where *H*: height, *W*: width, $C^{\prime }=3$ for RGB

Each image is passed through a preprocessing pipeline that includes normalization and resizing:


$$I_{\text{norm}}=\frac{I-\mu }{\sigma }$$

- μ, σ: mean and standard deviation used for normalization

**Global average pooling layer**

The feature maps are reduced via global average pooling:


$$x_{\text{gap}}=\frac{1}{N}\sum_{i=1}^{N}X_{i}$$

- *N*: number of spatial elements in the feature map

This yields a fixed-size feature vector suitable for fully connected layers.

**Dense (Fully Connected) Layer with ReLU Activation**


$$h=\text{ReLU}(W_{1}x_{\text{gap}}+b_{1})$$

- $W_{1}\in {\mathbb{R}}^{k\times d}$: weights
- $b_{1}\in {\mathbb{R}}^{k}$: bias
- $h\in {\mathbb{R}}^{k}$: transformed hidden feature vector

**Output layer with Softmax**

Given *K* = 3 classes (BLB, BS, LB), apply Softmax:


$${\hat{y}}_{i}=\frac{\exp(W_{2}^{\left(i\right)}h+b_{2}^{\left(i\right)})}{\sum_{j=1}^{K}\exp(W_{2}^{\left(j\right)}h+b_{2}^{\left(j\right)})}$$

- ${\hat{y}}_{i}$: predicted probability for class *i*
- $W_{2}^{\left(i\right)},b_{2}^{\left(i\right)}$: weights and bias of output layer

**Loss function (categorical cross-entropy)**


$$ℒ=-\sum_{i=1}^{K}y_{i}\log({\hat{y}}_{i})$$

- *y*_*i*_: true class label (one-hot encoded)
- ${\hat{y}}_{i}$: predicted class probability

**Optimization**

Training uses SGD optimizer:


$$\theta \leftarrow \theta -\eta \cdot \nabla_{\theta }𝒞$$

- η: learning rate
- θ: model parameters
- 𝒞: cost function (typically cross-entropy loss)

**Feature extraction using DenseNet201**

Let:

- $f_{\theta }(\cdot )$: DenseNet201 model with pretrained weights θ
- $X=f_{\theta }(I_{\text{seg}})\in {\mathbb{R}}^{d}$: Extracted deep feature vector after global average pooling

DenseNet connects each layer *l* to every previous layer:


$$x_{l}=H_{l}([x_{0},x_{1},\ldots ,x_{l-1}])$$

- *H*_*l*_: non-linear transformation (BN → ReLU → Conv)
- [ ]: concatenation operator

**GrabCut segmentation (foreground isolation)**

The GrabCut algorithm models the problem as an energy minimization task:


E(L,θ,z)=U(L,θ,z)+V(L,z)

- *L*: binary label (foreground/background)
- θ: parameters of the Gaussian Mixture Models (GMMs)
- *z*: pixel values
- *U*: unary term (fit of pixels to GMMs)
- *V*: pairwise term (smoothness across neighboring pixels)

The output is a segmented image *I*_seg_ containing mostly the diseased region.

**Contour detection (disease region localization)**

Contours *C* are detected using image gradients:


$$G(x,y)=\sqrt{{\left(\frac{\partial I_{\text{seg}}}{\partial x}\right)}^{2}+{\left(\frac{\partial I_{\text{seg}}}{\partial y}\right)}^{2}}$$

Bounding contours are then localized:


C={(x,y)∣G(x,y)>T}

- *T*: threshold value used to isolate significant gradients

A pre-trained DenseNet201, with the base layers (4 dense blocks) frozen, is used to extract hierarchical features. In addition, a global average pooling layer and a softmax head (for 3 classes) were trained using SGD (lr = 0.001, 30 epochs). The GrabCut and contour detection methods used a deep learning approach with explainable segmentation to localize disease areas, which could help diagnose rice disease accurately.

Dense connections help preserve gradients and encourage feature reuse. The model demonstrates strong robustness and generalization abilities due to its high accuracy and low loss values. It maintained consistent performance across all testing phases by successfully eliminating overfitting and providing objective evaluation on unfamiliar data sets. The detection method, which combines dense feature extraction with segmentation features and contour recognition, reliably identifies rice leaf diseases. This system delivers precise disease detection by utilizing high spatial accuracy to locate and identify specific diseased regions for practical agricultural diagnostics.

#### ResNet152V2 architecture.

ResNet152V2, renowned for its residual learning, effectively addresses the vanishing gradient problem, enabling deep learning and performing well with highly complex images. The ResNet152V2 algorithm achieves superior rice disease classification by utilizing deep residual learning mechanisms for improved feature extraction and overcoming gradient vanishing issues. Complex patterns found in diseased leaves become more visible thanks to its deep architectural design, which enhances classification accuracy. The model’s efficient parameter optimization system delivers better generalization along with computational efficiency.


$$\begin{array}{cc} x_{\ell +1}=x_{\ell } & +{\text{Conv}}_{1\times 1}(\text{BN-ReLU}({\text{Conv}}_{3\times 3}(\\ & \text{BN-ReLU}({\text{Conv}}_{1\times 1}(\text{BN-ReLU}(x_{\ell }))))) \end{array}$$ (3)

The Equation 3 defines a pre-activational bottleneck block which exists within ResNet152V2. Xℓ enters three convolutional strata consisting of 1x1 followed by 3x3 followed by 1x1 layers which are batch normalized then rectified. A skip connection allows the addition of the final output to the original input (xℓ) which makes training of deep networks feasible.

**ResNet152V2 architecture**

Input image tensor:


$$x_{0}\in {\mathbb{R}}^{H\times W\times C}$$

Initial processing:


$$x_{1}=\text{MaxPool}({\text{Conv}}_{7\times 7}(x_{0}))$$

Residual block (basic unit):


$$x_{l+1}=x_{l}+F(x_{l},W_{l})$$

where *F* contains:

- BatchNorm → ReLU → Conv_1×1_
- BatchNorm → ReLU → Conv_3×3_
- BatchNorm → ReLU → Conv_1×1_

For dimension matching:


$$x_{l+1}={\text{Conv}}_{1\times 1}(x_{l})+F(x_{l},W_{l})$$

Final classification:


$$\hat{y}=\text{Softmax}(W\cdot \text{GlobalAvgPool}(x)+b)$$

**Fig 8:**
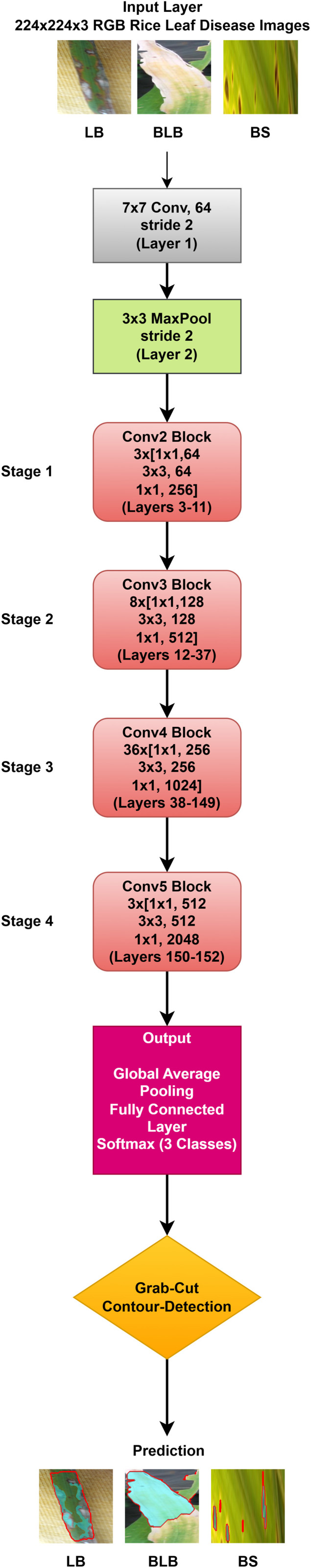
ResNet152V2 architecture with proposed solution.

The proposed solution integrates the ResNet152V2 model into a system that combines deep learning and contour-based segmentation for rice leaf disease classification, as shown in Fig 8. The process begins by dividing rice leaf images into different sections for training, validation, and testing. The ResNet152V2 model extracts deep hierarchical features from its 152 residual layers to improve both feature learning and gradient optimization. The input features pass through a global average pooling layer, followed by a dense ReLU layer for further processing. The softmax output layer provides high accuracy in disease classification as the final step. The system delivers excellent disease detection performance along with precise identification of the affected leaf regions. The segmentation process uses the extracted features from GrabCut to better separate diseased areas. The segmented output then undergoes contour detection to highlight disease-affected regions for improved visualization.

#### Mathematical model for the ResNet152V2 architecture with proposed solution.

**Input image representation**

Let each input image be represented as:


$$I\in {\mathbb{R}}^{H\times W\times C}$$

- *H*, *W*: image height and width
- *C* = 3: number of channels (RGB)

Image normalization:


$$I_{\text{norm}}=\frac{I-\mu }{\sigma }$$

- μ: mean, σ: standard deviation for normalization

**Global average pooling layer**

To convert feature maps into fixed-length vectors:


$$x_{\text{gap}}=\frac{1}{N}\sum_{i=1}^{N}X_{i}$$

- *N*: total number of spatial positions
- *x*_gap_: pooled feature vector

**Dense layer with ReLU activation**

The dense layer transforms features into class-representative space:


$$h=\text{ReLU}(W_{1}x_{\text{gap}}+b_{1})$$

- $W_{1}\in {\mathbb{R}}^{k\times d}$: weight matrix
- $b_{1}\in {\mathbb{R}}^{k}$: bias
- *h*: output from dense layer

**Output layer with Softmax**

For *K* = 3 classes (BLB, BS, LB):


$${\hat{y}}_{i}=\frac{\exp(W_{2}^{\left(i\right)}h+b_{2}^{\left(i\right)})}{\sum_{j=1}^{K}\exp(W_{2}^{\left(j\right)}h+b_{2}^{\left(j\right)})}$$

- ${\hat{y}}_{i}$: predicted probability for class *i*
- $W_{2}^{\left(i\right)},b_{2}^{\left(i\right)}$: output layer parameters

**Loss function (categorical cross-entropy)**


$$ℒ=-\sum_{i=1}^{K}y_{i}\log({\hat{y}}_{i})$$

- *y*_*i*_: one-hot encoded true label
- ${\hat{y}}_{i}$: predicted class probability

**Model optimization**

Using SGD optimizer:


$$\theta \leftarrow \theta -\eta \cdot \nabla_{\theta }𝒞$$

- η: learning rate
- θ: all learnable parameters in ResNet and dense layers

**Deep feature extraction using ResNet152V2**

Let $f_{\theta }(\cdot )$ denote the ResNet152V2 model. The key innovation of ResNet is the use of residual learning via identity shortcuts:

**Residual block equation:**


$$x_{l+1}=F(x_{l},W_{l})+x_{l}$$

- *x*_*l*_: input to layer *l*
- F(·): residual function (convolution, batch normalization, ReLU)
- $W_{l}$: weights of the layer

The output after full forward propagation is:


$$X=f_{\theta }(I_{\text{seg}})\in {\mathbb{R}}^{d}$$

- Deep feature vector extracted by ResNet152V2

**GrabCut segmentation (foreground isolation)**

GrabCut models segmentation using an energy minimization framework:


E(L,θ,z)=U(L,θ,z)+V(L,z)

- *L*: label assignment (foreground/background)
- θ: GMM parameters for color distributions
- *z*: pixel intensities
- *U*: data term (color model fit)
- *V*: smoothness term

Output: segmented image $I_{\text{seg}}\in {\mathbb{R}}^{H\times W\times C}$

**Contour detection**

Contours are derived from the edge gradients of the segmented image:


$$G(x,y)=\sqrt{{\left(\frac{\partial I_{\text{seg}}}{\partial x}\right)}^{2}+{\left(\frac{\partial I_{\text{seg}}}{\partial y}\right)}^{2}}$$

Contours:


C={(x,y)∣G(x,y)>T}

- *T*: edge threshold value

This step enhances localization of infected leaf areas.

#### EfficientNetV2L architecture.

EfficientNetV2L model is a convolutional neural network which uses an optimized scale of depth, width and resolution though the major architectural inventions are aimed at training efficiency. Our implementation makes use of an input image size of it is 384 x 384 as shown in the preprocessing pipeline. The choice was made as an effective tradeoff, since although EfficientNetV2L has been designed to accept 480 by 480, a downsampling to 384 by 384 would cut computational and memory costs greatly during both training and inference. This tradeoff is essential to the feasibility of hardware with limited resources, and at the same time yet the situation of the sufficient spatial detail to extract meaningful features of image.

The first stage of the network, which is also known as Stage 1, is set to capture low level spatial characteristics like edges and textures. It starts by providing a conventional convolution stage on the input image. This is then followed by a sequence of Fused-MBConv blocks. As opposed to the classic MBConv, the Fused-MBConv does not use expansion convolution and depthwise convolution but a 3x3 standard convolution operation. A convolutional operation in these blocks is succeeded by Batch Normalization and Swish activation function which is mathematically expressed as:


$$\text{Swish}(x)=x\cdot \sigma (x)=\frac{x}{1+e^{-x}}.$$

Swish can be used to alleviate the vanishing gradient issue and can be used to have a smoother optimization as the frozen base layers are being first trained.

The Stage 2 of the model then moves on to the extraction of high level and semantic features. This phase consists of regular MBConv blocks, that make use of depthwise separable convolutions in order to cut the cost of computing. An important element that is incorporated in these blocks is the Squeeze-and-Excitation (SE) module. SE module: The SE module conducts channel-wise attention, and processes the dimension of spatial information, which is represented as H W C, by initially squeezing it into one dimension (per channel) with Global Average Pooling (GAP):


$$z_{c}=F_{\text{sq}}(u_{c})=\frac{1}{H\times W}\sum_{i=1}^{H}\sum_{j=1}^{W}u_{c}(i,j).$$

This value, or descriptor, z, is then sent through two fully connected layers to produce a set of per-channel weights, the so-called excitation:


$$s=\sigma (W_{2}\cdot \text{ReLU}(W_{1}\cdot z)),$$

where *W*_1_ and *W*_2_ are learnable weights, and σ is the sigmoid function. The output *s* is a scaling factor that recalibrates the original feature map *u*_*c*_ by ${\tilde{x}}_{c}=s_{c}\cdot u_{c}$. This process enables the network to highlight informative features and ignore the less useful features. Another characteristic of this phase is a gradual increasing channel dimensions, which are ready to feature maps at the last classification layer.

After the MBConv stack of blocks, the feature maps are sent to the classification head. The Global Average Pooling is applied first to reduce a set of spatial dimensions to a 1x1 vector, which significantly reduces the number of parameters as compared to a fully connected layer and makes the model resistant to spatial translations. This is then passed through a fully-connected Dense layer. Afterwards, the logits are transformed to a probability distribution across the three disease target categories, with the help of the Softmax activation function. The equation of the Softmax of the class i is:


$$P(y=i|𝐳)=\frac{e^{z_{i}}}{\sum_{j=1}^{3}e^{z_{j}}},$$

and the value of the output of the preceding dense layer is the vector of values, denoted as the z. This gives the last probabilistic classification result of the model.

**Fig 9:**
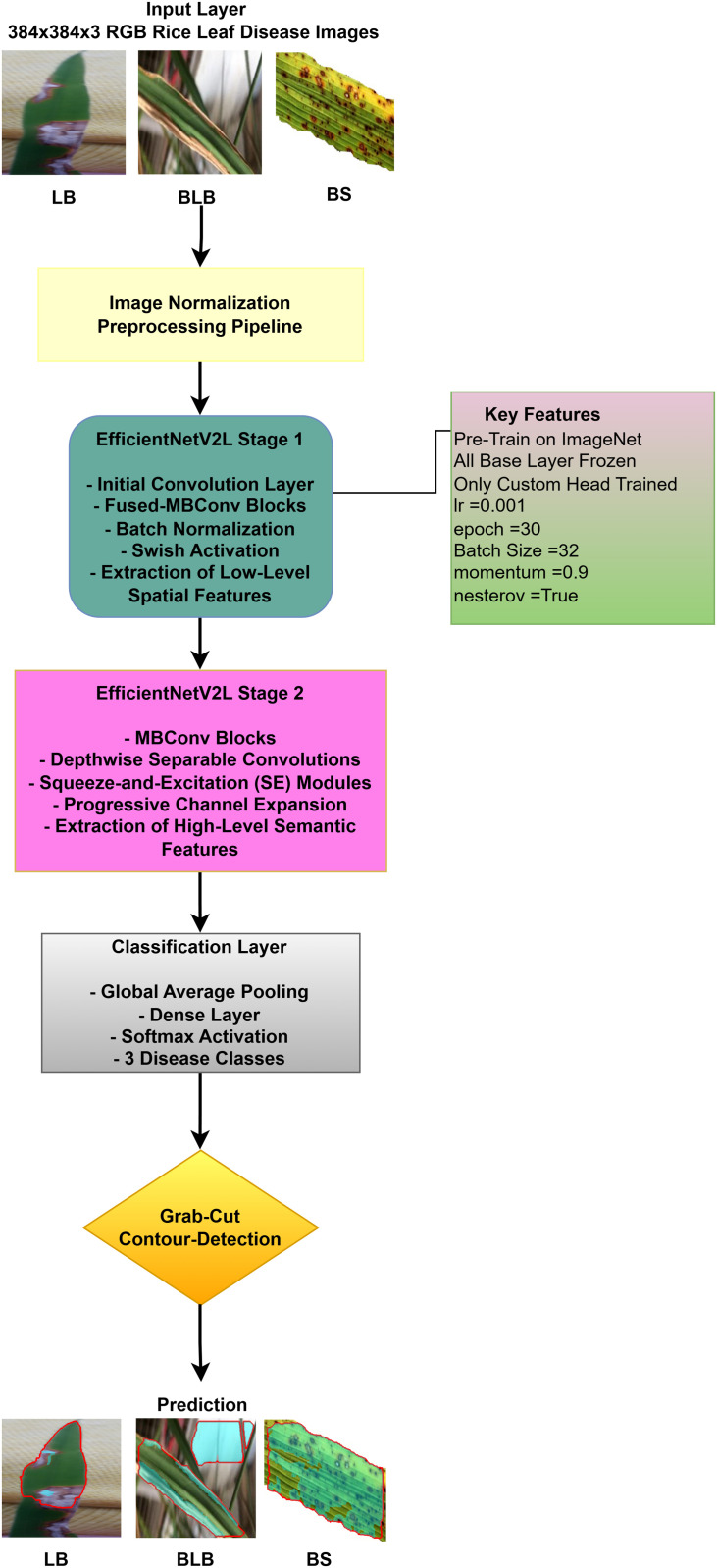
EfficientNetV2L Architecture with proposed solution.

The architecture of the rice leaf disease classification system using the EfficientNetV2L will be shown in the Fig 9. To start with the input images are normalized and preprocessed so as to maintain similarities between features. The EfficientNetV2L backbone is built on hierarchical features by using Fused-MBConv and MBConv blocks, which allow the acquisition of low-level spatial features and high-level semantic features. Lastly, the classification head with global average pooling and a softmax layer is predicted to predict the disease class and Grabcut segmentation with contour detection is use to outline the affected areas of the rice leaf.

#### MobileNetV2 architecture.

MobileNetV2 is an efficient convolutional neural network that is targeted at mobile and embedded vision applications. Its architecture is mostly founded on depthwise separable convolutions and reversed residual blocks made up of linear bottlenecks, that greatly lower the computational cost and provide the same power of representation.

Where the given input image is represented as:


$$x\in {\mathbb{R}}^{H\times W\times C}$$

In which H and W denote the spatial height and width of the feature map and C denotes the number of channels.

MobileNetV2 architecture The MobileNetV2 architecture starts with an expansion layer, which enlarges the input feature map dimensionality with a convolution size of 1 × 1:


$$f_{e}=\sigma \left(W_{e}*x\right)$$

In which *W*_*e*_ is the weights of the expansion convolution, * represents convolution, and σ(·) is the non-linear activation function (ReLU6).

Then, each channel is separately subjected to a depthwise convolution in order to obtain spatial features:


$$f_{d}=\sigma \left(W_{d}⊙f_{e}\right)$$

In which, *W*_*d*_ is the depthwise convolution kernel and ⊙ denotes channel-wise convolution.

A spatial feature is extracted after which, a linear projection (bottleneck layer) is used to minimize the dimensions of the feature map:


$$f_{p}=W_{p}*f_{d}$$

and the weights of the projection convolution are denoted by *W*_*p*_ and the non-linear activation is omitted so as to retain information in the low-dimensional space.

A residual connection is used when the input and output feature maps are of the same spatial and channel dimensions:


$$y=x+f_{p}$$

where *y* is the result of the inverted residual block output.

Therefore, the entire block transformation of MobileNetV2 can be summed up as:


$$y=x+W_{p}*\sigma \left(W_{d}⊙\sigma \left(W_{e}*x\right)\right)$$

It is such formulation that allows MobileNetV2 to attain high accuracy with a much smaller number of parameters and computation processes than the conventional convolutional neural networks.

**Fig 10:**
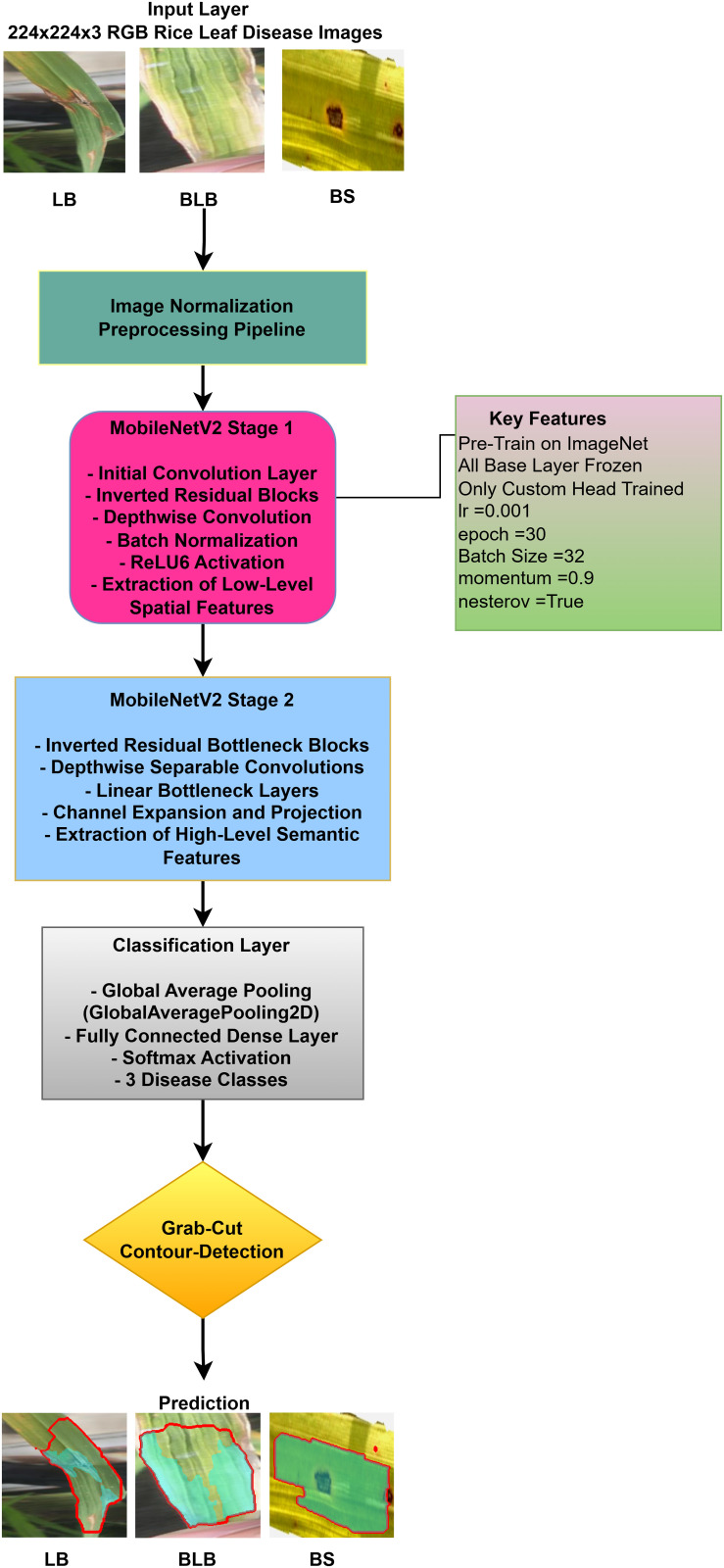
MobileNetV2 Architecture with proposed solution.

In this Fig. 10, a two-step pipeline of the MobileNetV2 based on the classification of plant diseases is described starting with frozen, pre-trained layers which extract low-level spatial features, then inverted residual block layers that can be trained to extract high-level semantic features. The architecture ends with a global average pooling layer and a softmax-activated dense layer to classify three types of diseases. MobileNetV2 transfer learning model with Grab-Cut contour detection identify disease region is used to train the model with hyper parameters like learning rate of 0.001, momentum 0.9 and Nesterov acceleration of more than 30 epochs.

## Research procedure and flowchart algorithm

The process of researching rice leaf disease classification with InceptionV3, DenseNet201, ResNet152V2, EfficientNetV2L and MobileNetV2 begins by loading and augmenting image datasets using methods like rotation, zoom, and flip to increase diversity. Images are resized to fit the input parameters of each CNN image 299 × 299 for InceptionV3). Pre-trained models (InceptionV3, DenseNet201, ResNet152V2, EfficientNetV2L and MobileNetV2) are employed with frozen convolutional layers to utilize transfer learning. Custom classification layers, including Global Average Pooling, dense layers, and dropout, are added for fine-tuning. These models are trained using categorical cross-entropy loss and the SGD optimizer. Training involves learning rate scheduling and model checkpointing. Finally, the models are evaluated on validation and test datasets, with accuracies reported.

**Fig 11:**
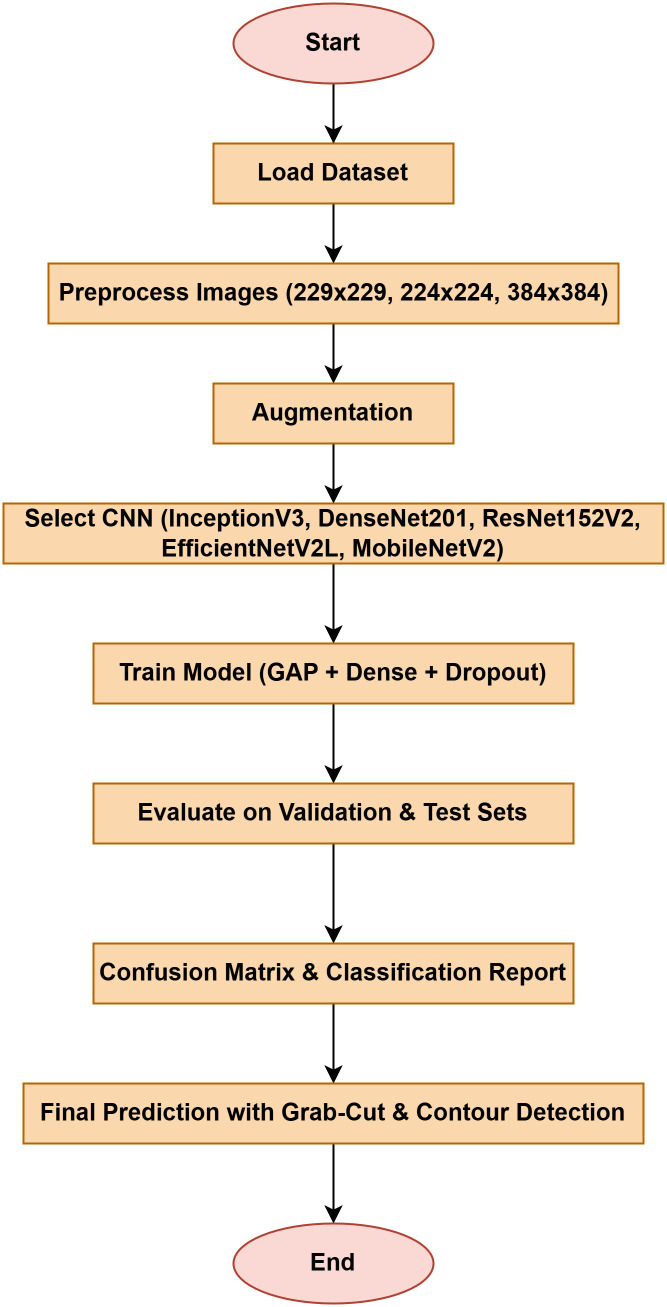
Flowchart of the proposed Enhanced Rice Leaf Disease Classification via Contour-Driven Segmentation and Optimized Deep Transfer Learning Architectures.

The flowchart shown in Fig 11 presents a proposed rice leaf disease classification framework based on deep transfer learning. First, it loads the labeled rice leaf dataset and then preprocesses the images to a uniform size. Data augmentation methods are subsequently applied only to the training set to improve the model’s generalization. The user can then choose one of three CNN architectures InceptionV3, DenseNet201, ResNet152V2, EfficientNetV2L and MobileNetV2 to be used for feature extraction and classification. The selected model is trained within a structure that includes a Global Average Pooling (GAP) layer, a Dense layer, and Dropout for regularization. The trained model is evaluated using the test and validation sets. Performance assessment is conducted through confusion matrices and a classification report. Finally, the model is used to predict disease types in unseen rice leaf images. This systematic approach provides robust and accurate detection of disease symptoms. The final prediction involves GrabCut and contour detection for foreground leaf segment identification, along with line extraction for disease localization.

## Explicit algorithm block

**Algorithm 1 Algorithm of proposed Enhanced Rice Leaf Disease Classification via Contour-Driven Segmentation and Optimized Deep Transfer Learning Architectures**

1: **Input**: Rice leaf image acquisition

2: **Preprocessing**: Image resizing and normalization

3: **CNN Inference**: Forward pass through deep network

4: **Probability Mapping**: Softmax activation for pixel-wise probabilities

5: **Class Prediction**: Argmax operation for class index determination

6: **ROI Initialization**: Automatic GrabCut rectangle generation

7: **Segmentation Refinement**: Iterative foreground-background separation

8: **Color Space Conversion**: RGB to HSV transformation

9: **Thresholding**: Lesion pixel isolation using HSV bounds

10: **Boundary Detection**: Contour extraction and localization

11: **Output**: Final prediction with segmented disease region mask

## Results and findings

In this research, InceptionV3, DenseNet201, ResNet152V2, EfficientNetV2L and MobileNetV2 are the transfer learning models used for rice leaf disease detection with balanced data. The dataset consisted of three classes: Brown Spot, Leaf Blast, and Bacterial Leaf Blight, with each class, there were 446 training images, 128 validation images, and 64 test images. Accuracy was selected as the primary evaluation criterion because it best reflected this middle situation. Then, more advanced filters, GrabCut and contour detection methods, were applied to the images to improve classification and visualization of diseased areas for each class. These methods achieved high accuracy in disease classification and differentiation, demonstrating the models’ effectiveness in practical use.

To ensure fair comparison across architectures, all baseline models (InceptionV3, DenseNet201, ResNet152V2, EfficientNetV2L, MobileNetV2) were trained under identical conditions, including the same dataset splits, augmentation pipeline, optimizer (SGD), learning rate (0.001), batch size (32), and number of epochs (30). No model received preferential hyperparameter tuning.

### Performance metrics

The decision to use accuracy as the performance metric in our study is justified because our dataset demonstrates perfect balance between classes, which highlights accuracy as a suitable diagnostic tool for measuring overall model performance. A balanced class distribution in datasets makes precision, recall, and F1-score less useful for performance evaluation, as results are not influenced by distribution patterns. The accuracy measurement allows us to observe how well the model detects rice leaf diseases across all disease classes.

#### Confusion matrix.

The confusion matrix helps users gain comprehensive analyses of model prediction outcomes through its presentation of TP, TN, FP, and FN values. The simple integration between precision and recall provides unclouded outcomes for evaluating the model without complexifying its analysis.

#### Accuracy.

The performance metric Accuracy determines how many instances a model correctly categorizes when compared to the complete dataset. When used on balanced data sets the accuracy measurement demonstrates how well the model performs regarding correct predictions. The formula for accuracy is:


$$\begin{array}{cc} \text{Accuracy} & =\frac{\text{TP}+\text{TN}}{\text{TP}+\text{TN}+\text{FP}+\text{FN}} \end{array}$$ (4)

Where: in Equation 4, Accurate predictions of positive samples are known as TP (True Positive). TN shows correct identification of negative cases. The classification of instances as positive turns out to be incorrect when performing predictions. False positives occur in this scenario. An incorrect assessment of negative examples leads to FN (False Negative).

#### Precision.

In Equation 5, precision is the ratio between the number of positive instances correctly predicted (True Positives) and all the instances predicted to be positive (True Positives + False Positives). It determines the accuracy of positive predictions:


$$\text{Precision}=\frac{TP}{TP+FP}$$ (5)

#### Recall.

Equation 6, recall indicates the accuracy of the proportion of positive cases predicted correctly (Positively predicted instances: True Positives) against all actual positive cases (True Positives + False Negatives). It tests the effectiveness of the model in picking up all possible cases.


$$\text{Recall}=\frac{TP}{TP+FN}$$ (6)

#### F1-score.

Equation 7, the F1-score combines Precision and Recall using a harmonic mean to produce a balanced measure of the two values. It can be applied in the case of unequal distribution of classes.


$$F_{1}=2\cdot \frac{\text{Precision}\cdot \text{Recall}}{\text{Precision}+\text{Recall}}$$ (7)

### InceptionV3 evaluation

In the proposed article, the InceptionV3 model is used to evaluate the concept of transfer learning. For the training set, the dataset is prepared using the following data augmentation: rescaling, shear, zoom, and horizontal flipping; for the validation and test sets, only rescaling is applied. The implementation begins with InceptionV3 serving as a base model, which lacks the softmax layer, so its final convolutional layer becomes the feature extractor during fine-tuning. A new classification head includes three layers: global average pooling, followed by a dense layer with 1024 neurons, and a softmax layer that predicts disease classes.

The pre-trained weights of the base model remain frozen during execution while the model is compiled with the SGD optimizer and categorical cross-entropy loss. The training process involves implementing early stopping and model checkpoint callbacks to prevent overfitting and ensure optimal results. The classification task includes three disease categories, represented by three output neurons corresponding to Brown Spot, Leaf Blast, and Bacterial Leaf Blight.

**Fig 12:**
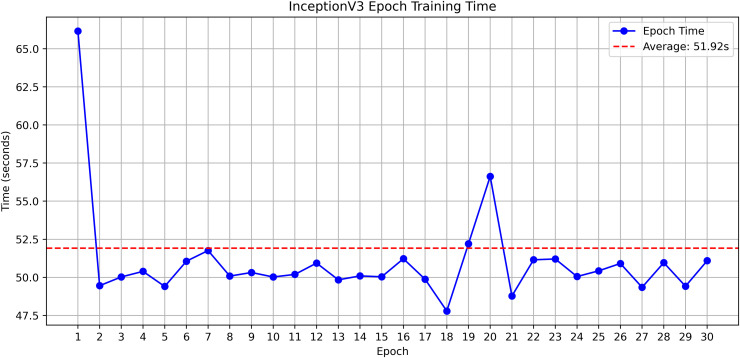
InceptionV3 model epoch training time.

InceptionV3 model on a batch size of 32 with GPUs of configuration NVIDIA T4×2 took an average of epoch-time of 51.92 seconds (i.e., 25.96 minutes to complete 30 epochs) with a learning rate of 0.001 as shown in Fig 12.

**Fig 13:**
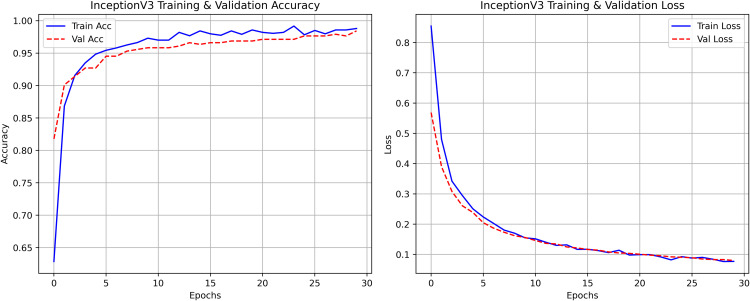
InceptionV3 training & validation accuracy, loss.

Hence, the output layer is defined based on testing performance on a test set over 30 epochs of training; metrics such as training accuracy and validation accuracy are measured, which constitute the results. Fig 13 shows the accuracy and loss plotted across epochs for training and validation, to analyze the InceptionV3 model’s performance trend. The developed model achieved 98.80% training accuracy and 98.44% validation accuracy, along with 98.43% test accuracy, indicating high predictive accuracy with minimal loss during training and validation. The model demonstrated strong generalization capabilities while avoiding overfitting throughout training.The model exhibits strong learning performance that shows steady improvement and high levels of robustness which make it suitable in rice leaf disease classification.

**Fig 14:**
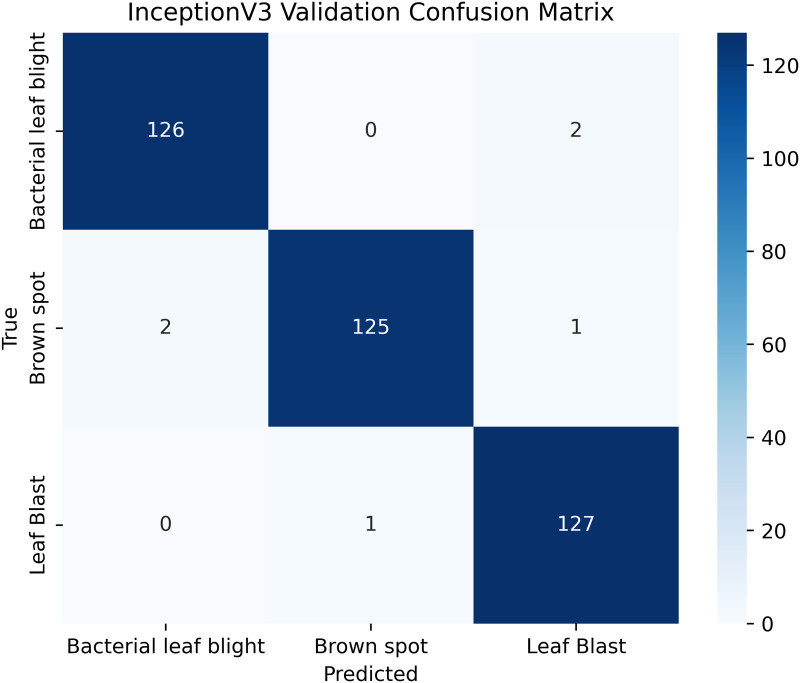
InceptionV3 model validation confusion matrix.

The confusion matrices display how InceptionV3 performs in validating and testing phases of rice leaf disease classification. The validation confusion matrix indicates a little misclassification errors, with the Bacterial leaf blight predicted as Leaf Blast in 2 cases, Brown spot misclassified in 2 instances as Bacterial leaf blight and another 1 as Leaf Blast, and 1 prediction of Leaf Blast as Brown spot revealing almost perfect discrimination of classes as shown in Fig 14.

**Fig 15:**
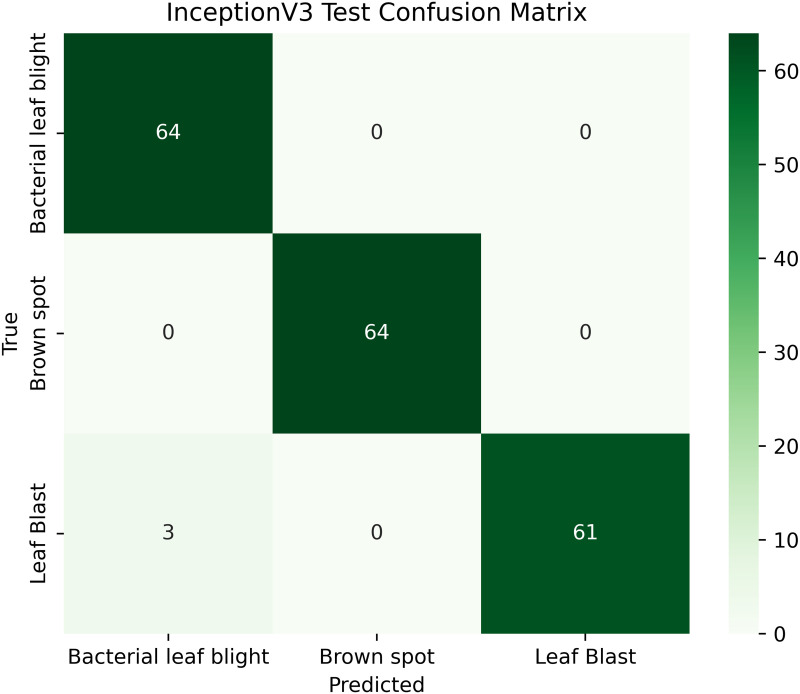
InceptionV3 model test confusion matrix.

The test confusion matrix indicate misclassified 3 instances of “Leaf Blast” as Bacterial Leaf Blight, as shown in Fig 15.

**Fig 16:**
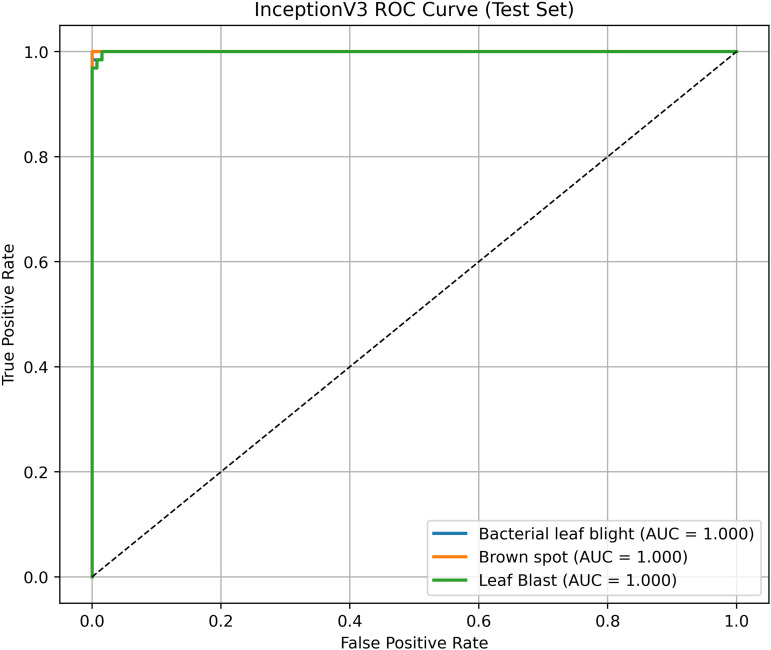
InceptionV3 model ROC Curve (Test Set).

InceptionV3 model exhibits superior performance in terms of classifications where ROC curves illustrate almost flawless discrimination of each of the three disease categories. The model has a value of 0.998 in terms of AUC, which means that there are only a few false positives, and the model is very much capable of classifying this type of bacteria among the rest. Brown spot and Leaf blast also obtain AUC of 0.997 and 0.998 respectively and indicates the excellent performance of the model in ranking positive correctly before the negative ones regardless of the threshold value, and confirms the strong test accuracy of the model was 98.43 percent as shown in Fig 16.

The Table 5 shows the performance of the model on the test set which was an accuracy of 98.43 percent. It presents good precision, recall, and F1-scores (0.96–1.00), in all 3 classes (Bacterial leaf blight, Brown spot, Leaf Blast), which means a balanced and effective prediction.

**Table 5:** InceptionV3 model classification report on test set.

Class	Precision	Recall	F1-score	Support
Bacterial leaf blight	0.96	1.00	0.98	64
Brown spot	1.00	1.00	1.00	64
Leaf Blast	1.00	0.95	0.98	64
**Test Accuracy**	—	—	0.9843	192
**macro-average**	0.99	0.98	0.98	192
**weighted-average**	0.99	0.98	0.98	192

To present our results, the InceptionV3 model, which was pre-trained on ImageNet, is applied to a set of randomly selected rice leaf disease images from the test set. Grab Cut segmentation is used to isolate the area of interest. The process involves obtaining images, preprocessing them by resizing and normalizing their shapes, and then classifying each image with associated confidence scores. For each image, Grab Cut is applied to segment the region, highlighting contours with a bluish color. The original images and the images with detected contours are displayed side by side using Matplotlib in two subplots.

**Fig 17:**
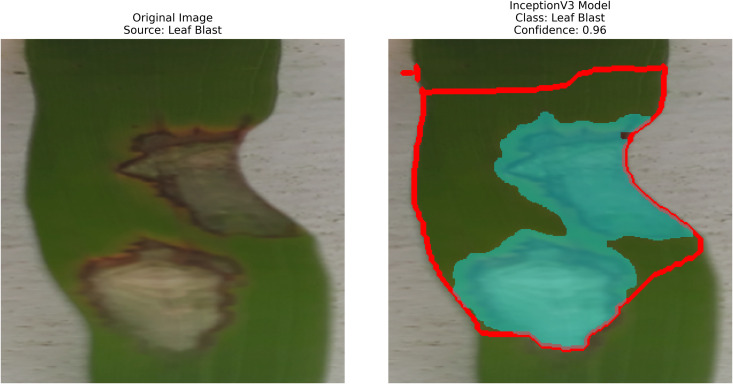
InceptionV3 leaf blast prediction.

**Fig 18:**
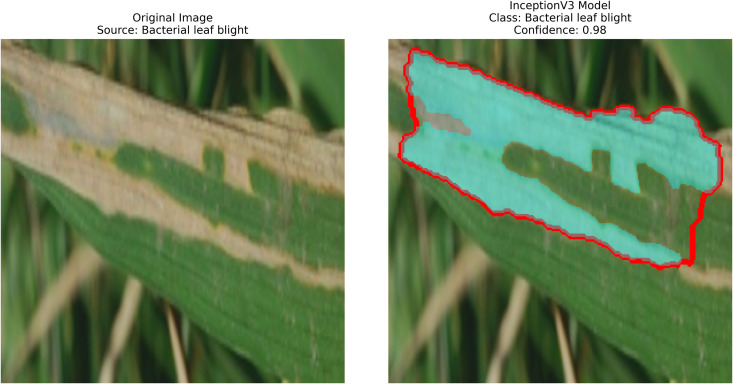
InceptionV3 bacterial leaf blight prediction.

**Fig 19:**
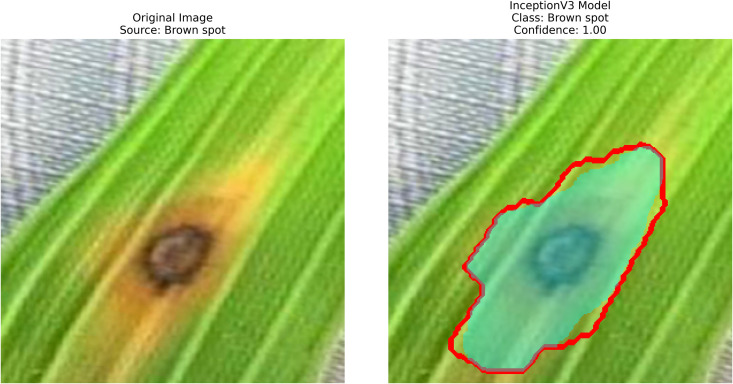
InceptionV3 brown spot prediction.

Fig 17 shows the results used to predict the presence of Leaf Blast disease on rice leaves using GrabCut for segmentation outlines. It is then processed for accurate disease classification, highlighting the blueish contours in the processed output alongside the original image with confidence 0.96. The results in Fig 18 demonstrate that the model can accurately identify Bacterial Leaf Blight and locate the affected areas on the leaves with confidence 0.98. Similarly, Fig 19 presents the results for Brown Spot disease, where the affected regions are effectively overlaid with contours on the map. These visualizations reflect the model’s ability to classify diseases and perform region-specific segmentation with highlighted contours and with confidence 100 percent.

### DenseNet201 evaluation

Trained a rice leaf disease classification model using DenseNet201 as the base. It sets up data generators for training, validation, and testing, applies augmentation to the training data, and scales all images to the input size 224*x*224. The DenseNet201 backbone is pre-trained on the ImageNet dataset, and the initial layers are frozen to prevent further learning; only classification layers are added on top of the DenseNet201 base, ending with softmax layers to estimate probabilities for three classes. The model is optimized using the SGD optimizer and trained for 30 epochs; early stopping and checkpointing are used to save the best model. Finally, the model is evaluated on the test set, and training and validation accuracies are reported.

**Fig 20:**
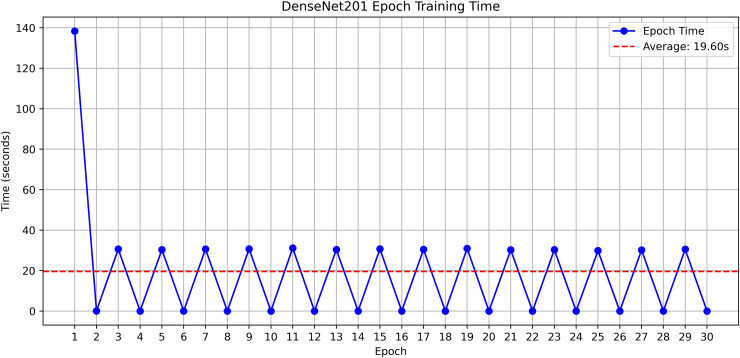
DenseNet201 model epoch training time.

The DenseNet201 model was trained with a batch size of 32 on a dual NVIDIA T4 GPU setup to ensure efficient high-performance computation. It took the model 19.60 seconds average epoch time to train, demonstrating the model’s efficiency in computing each iteration. Over 30 epochs, the total training time amounts to 9.80 minutes, indicating scalable training performance for the target task as shown in Fig 20.

**Fig 21:**
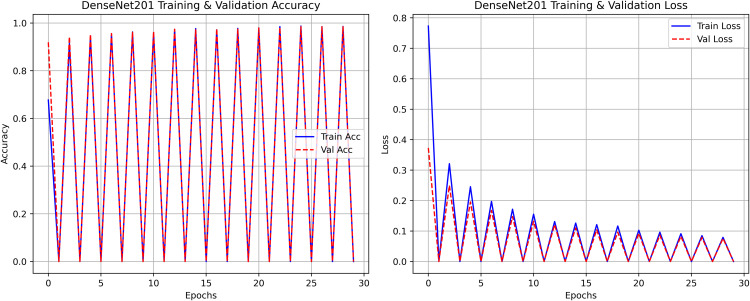
DenseNet201 model training & validation accuracy, loss.

The training and validation accuracy along with loss patterns for the DenseNet201 model during training are shown in Fig 21. The DenseNet201 model achieved high performance by reaching 98.72% training accuracy, followed by 98.43% validation accuracy and 98.43% test accuracy, indicating high classification accuracy with minimal loss. The model demonstrated strong learning ability without significant overfitting. It shows superior generalization because its accuracy metrics remain consistent across different datasets. The model reached unstable convergence with a learning rate of 0.001. During training, DenseNet201 model showed fluctuations in both training and validation accuracy graph, indicating inconsistent in convergence.

**Fig 22:**
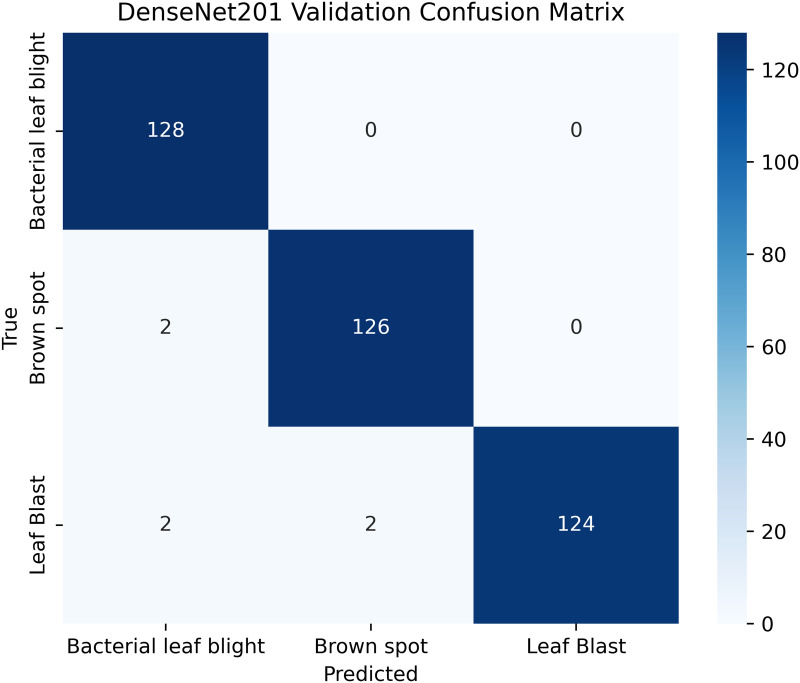
DenseNet201 validation confusion matrix.

The DenseNet201 model showed a few misclassifications on the validation set, as shown in Fig 22. Specifically, two instances of “Brown Spot” was incorrectly predicted as “Bacterial Leaf Blight.” Additionally, “Leaf Blast” was misclassified twice as “Bacterial leaf blight” and two as “Brown Spot.”

**Fig 23:**
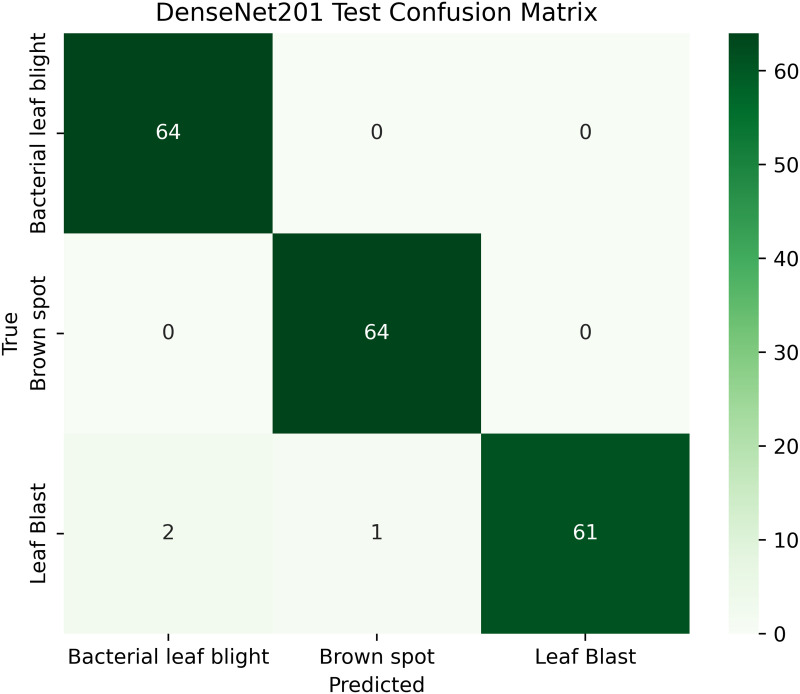
DenseNet201 model test confusion matrix.

The DenseNet201 model demonstrated strong performance on the test set with very few misclassifications as shown in Fig 23. All instances of “Bacterial leaf blight” and “Brown spot” were correctly identified. The only misclassifications occurred for “Leaf Blast,” where two instances were incorrectly predicted as “Bacterial leaf blight” and one instance predict as “Brown Spot.”

**Fig 24:**
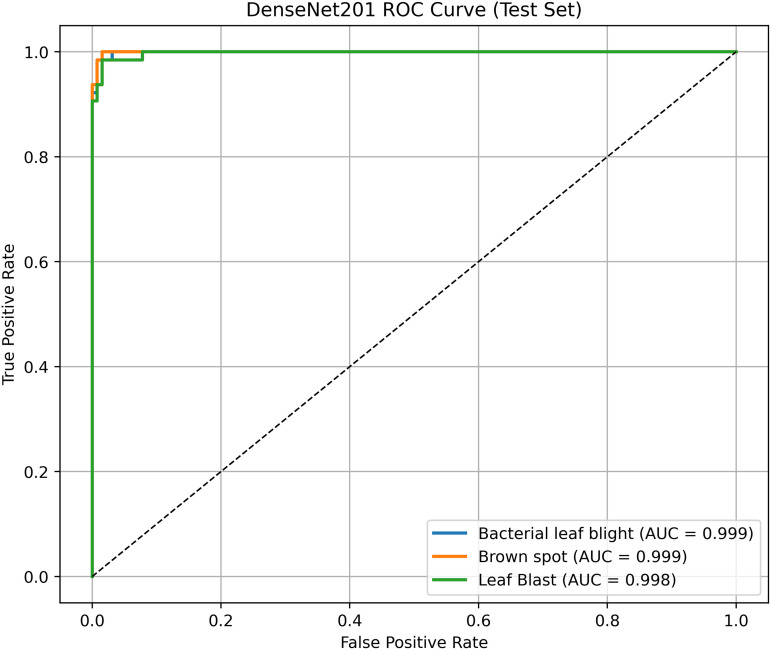
DenseNet201 model ROC Curve (Test Set).

DenseNet201 model has excellent discriminative power against all three classes of rice diseases with the ROC analysis indicating close to perfect performance in classification. The AUC of bacterial leaf blight is 0.997, which shows that the model is highly effective in separating this disease among other diseases with very few false positives. The same thing happens with brown spot and Leaf Blast where they report AUC of 0.998 and 0.997 respectively as shown in Fig 24, which is an affirmation that DenseNet201 always puts positive examples ahead of the negative ones at every classification threshold. Although DenseNet201 model showed good final test accuracy of 98.43, it demonstrated unreliable learning curves throughout the training period and this is demonstrated by noticeable oscillations in the accuracy and loss curve which showed inconsistent convergence behavior.

The model achieves exceptional performance with 98.43% test accuracy, demonstrating near-perfect precision, recall, and F1-scores (0.97–1.00) across all disease classes (Bacterial leaf blight, Brown spot, Leaf Blast), confirming robust and balanced classification capability suitable for real-world agricultural diagnostics as detail in Table 6.

**Table 6:** DenseNet201 model classification report on test set.

Class	Precision	Recall	F1-score	Support
Bacterial leaf blight	0.97	1.00	0.98	64
Brown spot	0.98	1.00	0.99	64
Leaf Blast	1.00	0.95	0.98	64
**Test Accuracy**	—	—	98.43	192
**macro-average**	0.98	0.98	0.98	192
**weighted-average**	0.98	0.98	0.98	192

**Fig 25:**
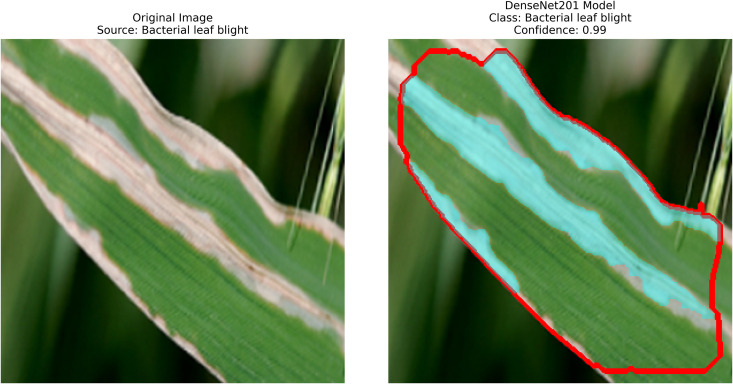
DenseNet201 bacterial leaf blight prediction.

In Fig 25, the predicted image compares with the original image of a rice leaf. Then, with a red outline GrabCut segmentation with bluish highlighted contours drawn, the expected image shows the affected region, which signals a Bacterial Leaf Blight. This prediction would be with 0.99 confidence, meaning we’re sure about it.

**Fig 26:**
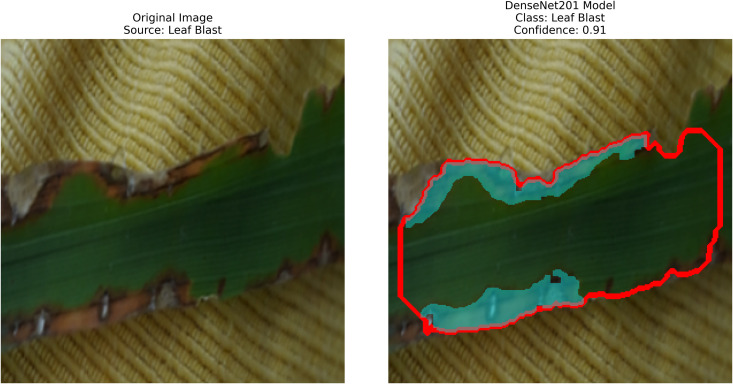
DenseNet201 leaf blast prediction.

A DenseNet201 deep learning model analyzed an image that the classification system initially identified as leaf blast disease in plants. Using the combination of the GrabCut technique with contour detection methods, the model generated its expected output. A red outline indicates the affected area in the predicted image, with bluish contours marking the regions of damage. The model expressed full confidence in its analysis by labeling the identified area as “Leaf Blast” with a confidence level of 91%, as shown in Fig 26.

**Fig 27:**
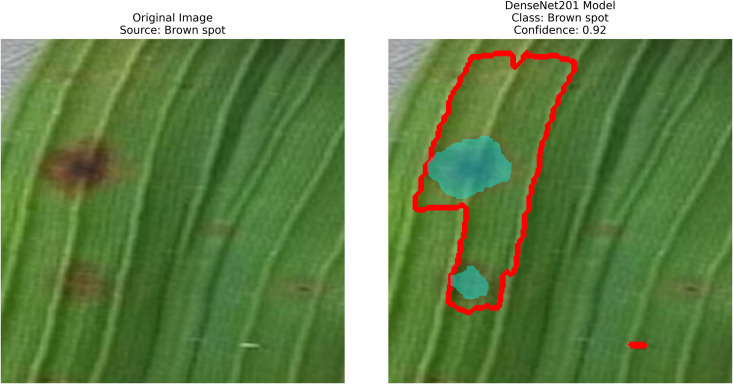
DenseNet201 brown spot prediction.

The segmentation of leaves affected by “Brown spot” is performed using GrabCut with contour detection, as shown in Fig 27. The original image is on the left, while the predicted image is on the right. In the predicted image, the GrabCut segmentation is outlined in red, effectively isolating the diseased area. A bluish-highlighted region displays detected contours, which help define the Brown spot boundary. DenseNet201 predicted the affected area as the Brown spot with 92% confidence.

### ResNet152V2 evaluation

Classifying rice leaf disease images systematically by extracting the contours of damaged areas using GrabCut segmentation. It extracts and classifies images with pre-trained ResNet152V2 models, ensuring each image is resized to 224*x*224 and preprocessed into the proper format. The load_image function reads and prepares the image, and the predictor provides a class label along with a confidence score. Using GrabCut segmentation techniques, contours are isolated and extracted from the foreground of the leaf image. We then visualize our classifications side by side with segmented images and highlighted contours to provide clear visual validation or disease analysis.

**Fig 28:**
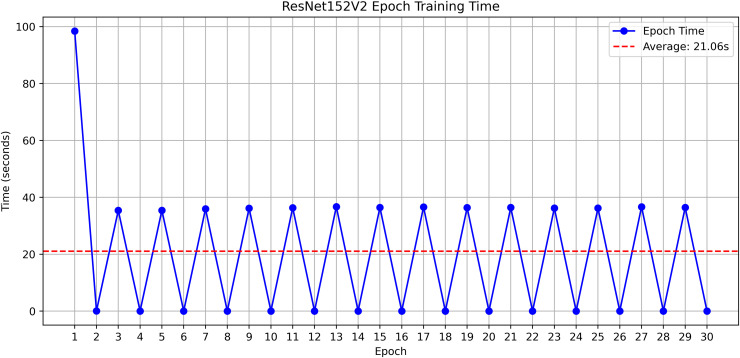
ResNet152V2 model epoch training time.

The training process of the ResNet152V2 model was configured with a batch size of 32 and used two NVIDIA T4 GPUs, which sped up the training and enhanced deep feature extraction. The provided graph in Fig 28, “ResNet152V2 Model Epoch Training Times (Total: 30 Epochs),” shows the time taken for each training epoch. The epoch times stabilize, averaging around 21.06 seconds per epoch for a total training time of 10.53 minutes over 30 epochs.

**Fig 29:**
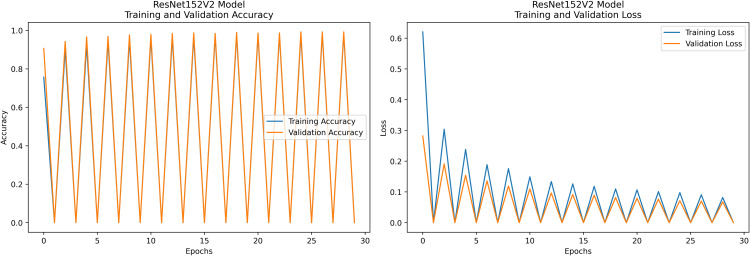
ResNet152V2 training & validation accuracy, loss.

As in Fig 29, results from testing the ResNet152V2 model indicated successful learning capabilities with training accuracy at 99.02%, validation accuracy at 99.22% and test accuracy is 97.39% with minimum loss rate. The model used 0.001 as its learning rate. In the training stage, ResNet152V2 exhibited serious fluctuations in accuracy as well as loss curves with successive epochs indicating unreliable convergence stability.

**Fig 30:**
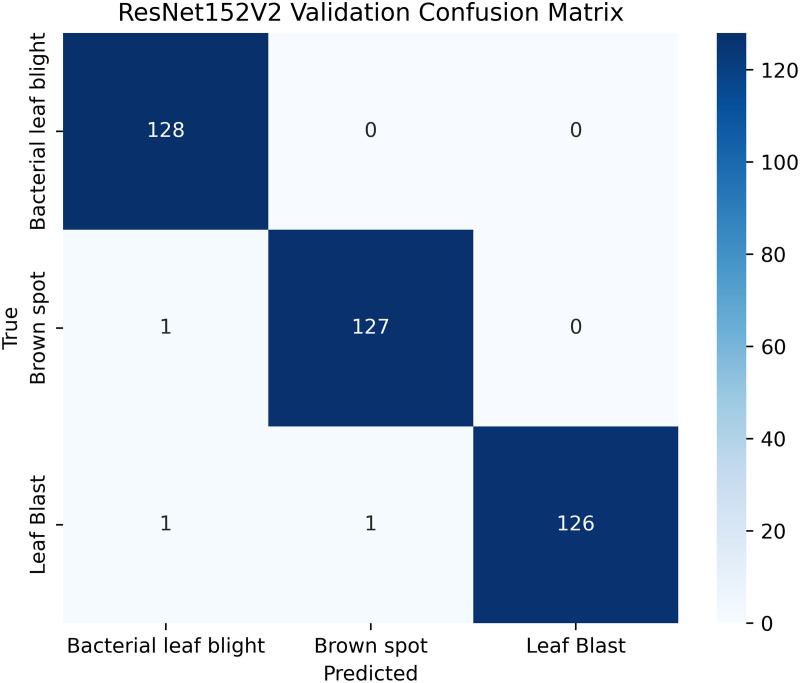
ResNet152V2 model validation confusion matrix.

The ResNet152V2 model showed very few misclassifications on the validation data. All instances of “Bacterial leaf blight” were correctly identified. “Brown spot” was incorrectly predicted as “Bacterial leaf blight.” Additionally, two “Leaf Blast” instances was mistakenly predicted as one time as “Bacterial leaf blight,” an other one is “Brown Spot” as shown in Fig 30.

**Fig 31:**
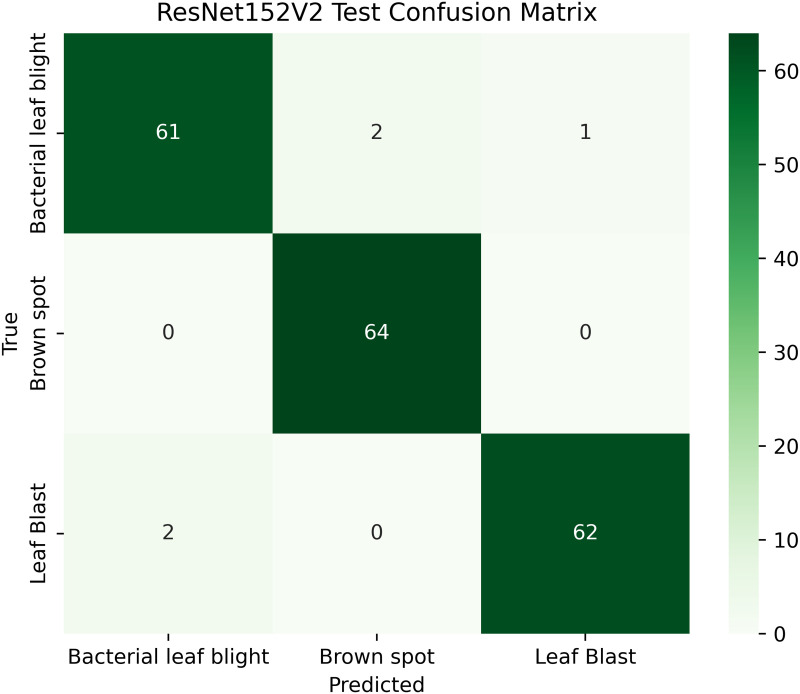
ResNet152V2 model test confusion matrix.

The ResNet152V2 model performed exceptionally well on the test set, with perfect classification for “Brown spot.” BLB 3 misclassifications (2 as Brown spot, 1 as Leaf Blast). Leaf Blast as 2 misclassifications both as Brown spot as shown in Fig 31. The model maintains good prediction accuracy across both evaluation sets, as the misclassifications highlight areas needing future improvement. Visualization color intensity indicates the distribution of images, with darker shades signifying a larger number of images.

**Fig 32:**
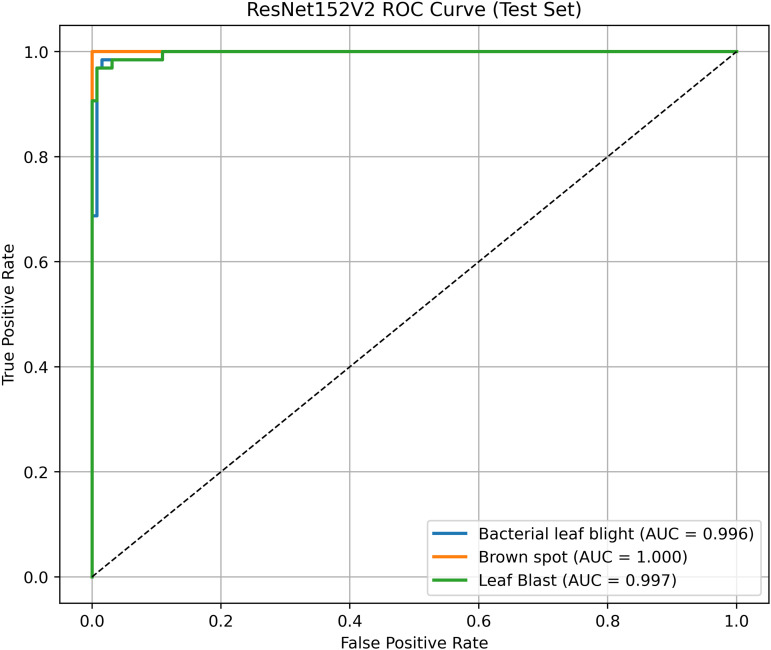
ResNet152V2 model ROC Curve (Test Set).

The ResNet152V2 model display an outstanding classification capability with Bacterial leaf blight, Brown spot, and Leaf Blast achieved AUC of 0.995, 0.998, and 0.996, as shown in Fig 32, suggesting that the model has an excellent discriminative capability on all the three types of diseases. The large values of the AUC indicate that the model always puts positive instances on top of negative ones with few false positives which is equivalent to high accuracy of the model shown to be 97.39. Although the ResNet152V2 model yields high AUC values in all classes, the model has severe oscillations throughout the training stage, unsteady convergence curves and inconsistent validation results show inconsistent learning behavior.

Table 7 describes the classification accuracy of a model on three classes of plant diseases with an outstanding test accuracy of 97.39 percent. According to the results the precision, the recall and the F1-scores are almost perfect (0.96−1.00) on all classes and the support is equal (64 samples per class) which suggests very reliable and consistent prediction.

**Table 7:** ResNet152V2 Model Classification Report on Test Set.

Class	Precision	Recall	F1-score	Support
Bacterial leaf blight	0.97	0.95	0.96	64
Brown spot	0.97	1.00	0.98	64
Leaf Blast	0.98	0.97	0.98	64
**Test Accuracy**	—	—	97.39	192
**macro-average**	0.97	0.97	0.97	192
**weighted-average**	0.97	0.97	0.97	192

**Fig 33:**
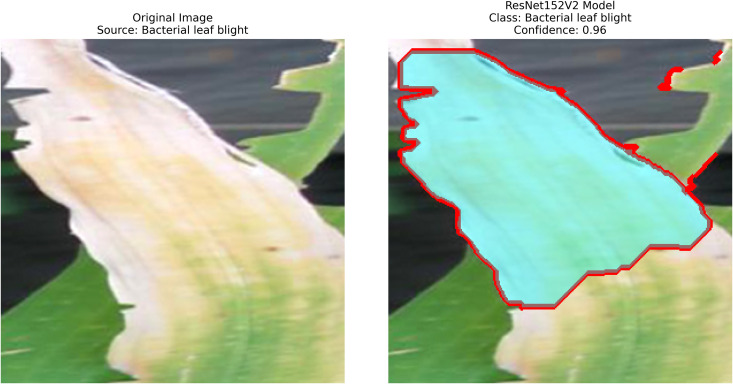
ResNet152V2 bacterial leaf blight prediction.

**Fig 34:**
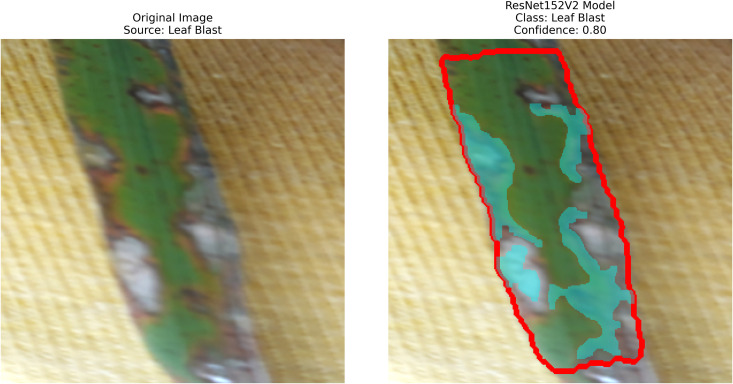
ResNet152V2 leaf blast prediction.

The ResNet152V2 model can segment and detect “Bacterial leaf blight” in plant leaf images, as shown in Fig 33. The original image displays a picture on the left, while the right image shows segmentation accuracy through GrabCut with a blue contour detection. The model attains a 96% confidence score in its classification of “Bacterial leaf blight” in the segmented area, reinforcing its ability to detect this particular disease. Using the ResNet152V2 model with GrabCut and contour detection, the system accurately detected and diagnosed “Leaf Blast” in the provided image. The original picture on the left shows the diseased area next to the processed version on the right, which highlights the disease region with red outlining from GrabCut segmentation and blue contours, as shown in Fig 34. The predicted segment is classified as “Leaf Blast” with a confidence level of 0.80, demonstrating accurate disease identification. The visual evidence illustrates how effectively the model detects and classifies plant diseases.

**Fig 35:**
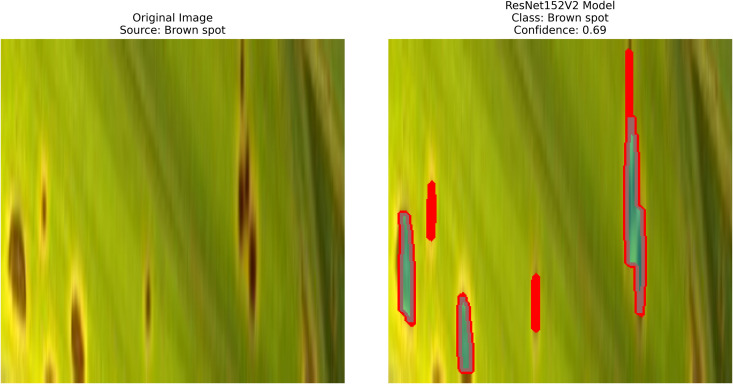
ResNet152V2 brown spot prediction.

By using the ResNet152V2 model combined with GrabCut and contour detection, the system successfully recognized and classified a leaf affected by a “Brown spot” disease in the image. The image on the left shows the original sample, while the one on the right displays the processed version marked with a red boundary around the “Brown spot” area. The model achieved an accuracy of 0.69 in detecting the distribution of “Brown spot” in the sectioned areas of the leaf, as shown in Fig 35.

### EfficientNetV2L evaluation

The process of evaluation starts with the preparation of the dataset with the help of directory-based generators loading training, validation, and testing images and implementing normalization and augmentation to enhance the process of generalization. The model is trained on an EfficientNetV2L backbone which is pre-trained, with frozen feature extraction layers and a three-class rice disease classification head of global average pooling and a softmax layer. Validation accuracy is checked during training process through a checkpoint mechanism that automatically saves the best-performing model and a custom-callback records the time taken per epoch. Once the training is done, the model performance is assessed on validation and test sets in terms of accuracy metrics and analysis of the confusion matrix and classification report to determine the effectiveness of the classification.

**Fig 36:**
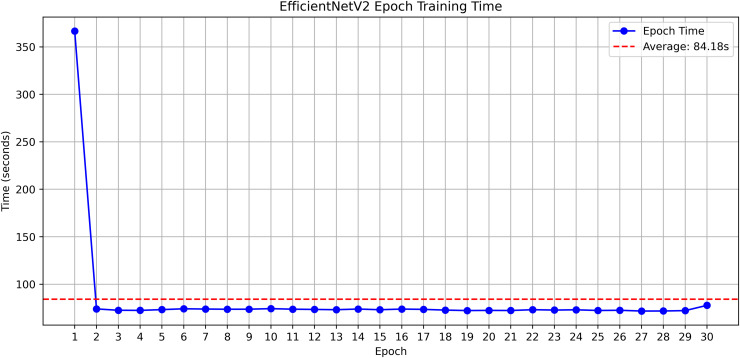
EfficientNetV2L model epoch training time.

The training of the model was done on a NVIDIA T4x2 system, and the entire process took 42.09 minutes, which showed an effective use of computational resources. The mean time of training per epoch was 84.18 seconds and this result shows Fig 36 the training process is stable and the computational complexity of the model is manageable.

**Fig 37:**
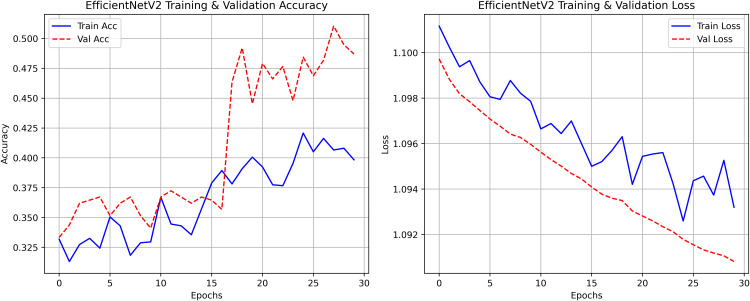
EfficientNetV2L training & validation accuracy, loss.

Fig 37 shows curves of the training reflect a divergent optimization direction, with the underperforming architecture (red) showing significantly lower accuracy and significantly higher loss values than that of the baseline (blue) and being unable to converge. This training dynamics is directly proportionate to the poor generalization of the model as shown by its a low training accuracy of 39.01% with a high loss of 1.0941, and a validation accuracy of 48.70% with a loss of 1.0908, which aligns with its poor test performance of 44.50%.

**Fig 38:**
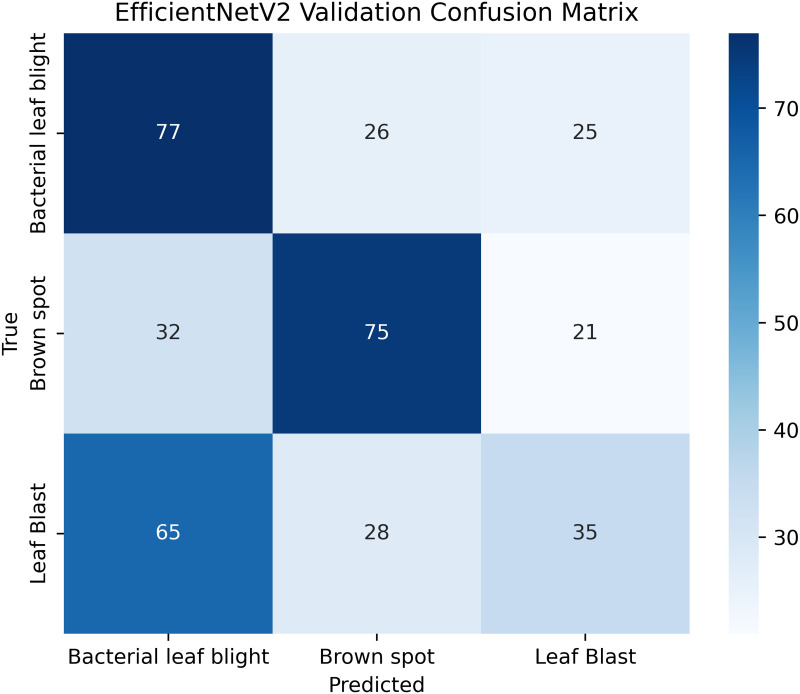
EfficientNetV2L model validation confusion matrix.

The confusion matrix Fig 38 shows the validation results of EfficientNetV2 reveal that the model has high confusion on all classes, and the most problematic one is Leaf Blast because 65 samples were mistakenly classified as Bacterial leaf blight and 28 samples as Brown spot. On the same note, there were 26 cases of Bacterial leaf blight that were mistaken to Brown spot and 25 to Leaf Blast and 32 cases of Brown spot that was predicted to be Bacterial leaf blight and 21 cases of Leaf Blast.

**Fig 39:**
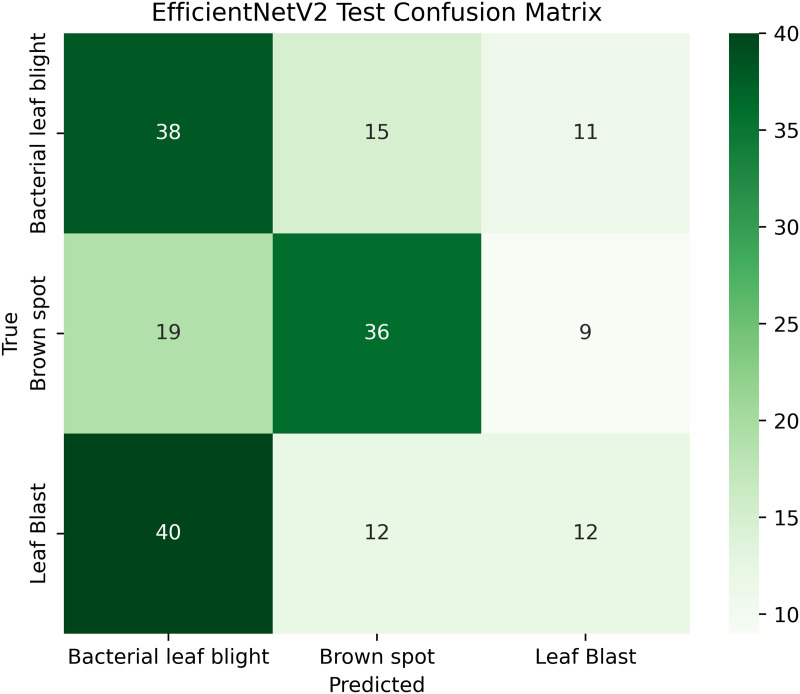
EfficientNetV2L model test confusion matrix.

The EfficientNetV2 model also had major problems with Leaf Blast, with 40 samples being wrongly labeled as Bacterial leaf blight and 12 as Brown spot, which means that only 12 samples were correctly predicted. Moreover, the Bacterial leaf blight was confused with Brown spot 15 times and Leaf Blast 11 times and 19 cases of Brown spot were falsely identified as Bacterial leaf blight and 9 cases as Leaf Blast as shown in Fig 39.

**Fig 40:**
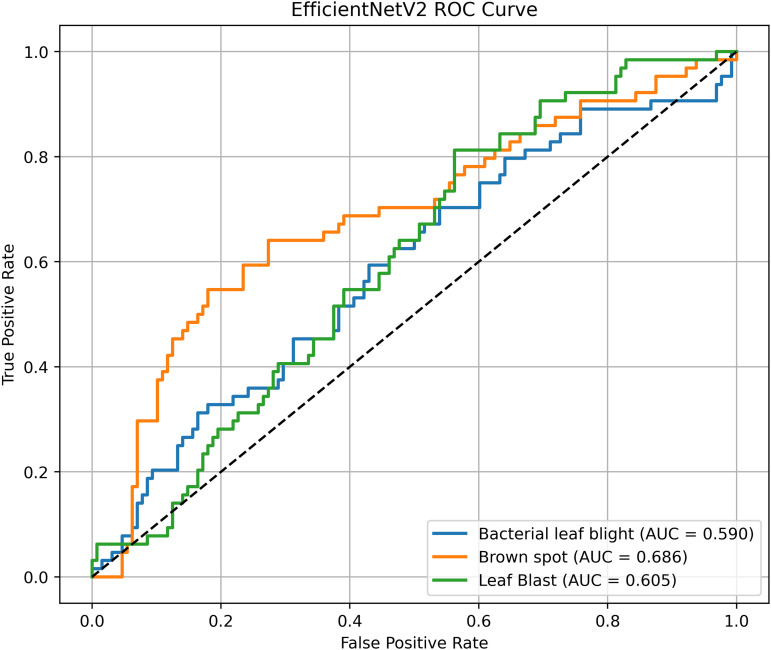
EfficientNetV2L model ROC Curve (Test Set).

The analysis of ROC curve as shown in Fig 40 indicates that Brown spot is the best at discriminative ability with an AUC of 0.686, then Leaf Blast with AUC of 0.605, and Bacterial leaf Blast with AUC of 0.590. All of these AUC values are relatively low, less than 0.70, which confirms the poor overall classification of the model and Brown spot is slightly better delineated than the other two disease classes.

Table 8 classification report would indicate a much lower model performance with a total accuracy rating of 45 per cent on an average and weighted averages of precision, recall and F1-score of between 0.43 to 0.45. In the individual classes, Brown spot had the highest F1-score of 0.57, whereas Leaf Blast had the lowest recall with the lowest F1-score of 0.25.

**Table 8:** EfficientNetV2L Model Classification Report on Test Set.

Class	Precision	Recall	F1-score	Support
Bacterial leaf blight	0.39	0.59	0.47	64
Brown spot	0.57	0.56	0.57	64
Leaf Blast	0.38	0.19	0.25	64
**Test Accuracy**	—	—	44.50%	192
**macro avg**	0.45	0.45	0.43	192
**weighted avg**	0.45	0.45	0.43	192

**Fig 41:**
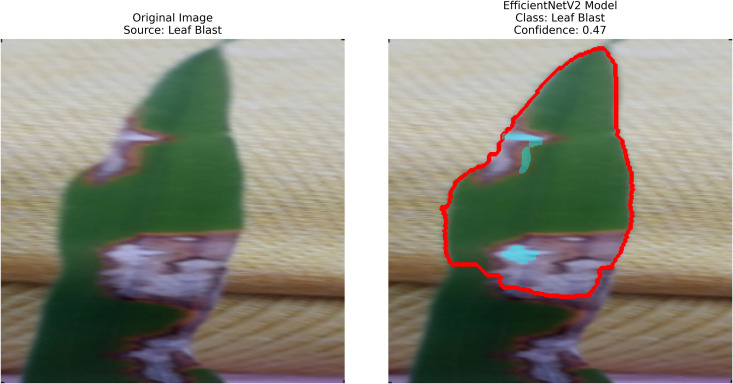
EfficientNetV2L leaf blast prediction.

**Fig 42:**
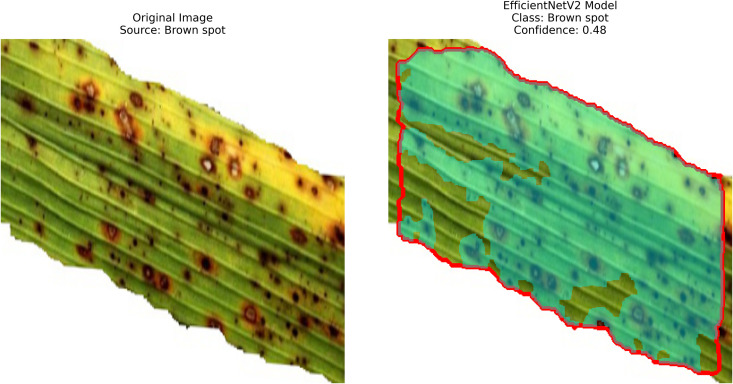
EfficientNetV2L Brown Spot prediction.

**Fig 43:**
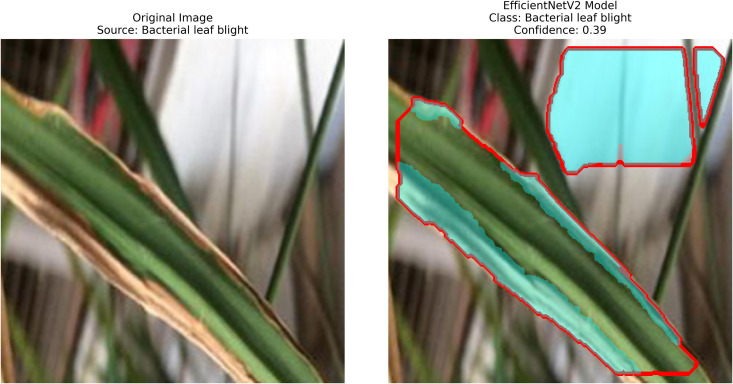
EfficientNetV2L Bacterial Leaf Blight prediction.

As shown in Fig 41 model identified the original image of Leaf Blast correctly, which indicated correct identification of the model on this category. The less confidence score of 0.47, however, is the only indication that there is a lot of uncertainty in its prediction. The Fig 42 shown prediction of the Brown Spot by the model has the confidence value of 0.48 with the least confidence distribution among the parameters that were analyzed and the segmentation of image using GrabCut with the contour detection identify region of disease.Fig 43 model recognised Bacterial Leaf Blight with a low confidence score of 0.39 which means that the classification was accurate but the decision boundary under this prediction was weak and the model showed great uncertainty when it comes to making a distinction about the disease-related features of surrounding rice leaf image.

### MobileNetV2 evaluation

Model establishes a MobileNetV2-based pipeline of transfer learning to classify the rice leaf diseases, paths to the datasets, preprocessing, and augmentation. It trains the base freezes-layers and prependes a classification head of their own and compiles using SGD and categorical cross-entropy. Training is executed with validation check, epoch time and model checkpointing to store the optimal weights.

**Fig 44:**
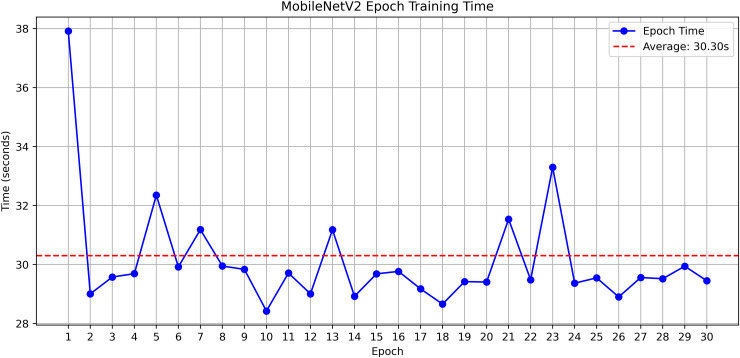
MobileNetV2 model epoch time.

Fig 44 shows entire time taken to train the model was 30 epochs and this took 15.15 minutes, equivalent to 30.30 seconds per epoch on the T4x2 GPU configuration. This means that there is a steady training rate in all epochs.

**Fig 45:**
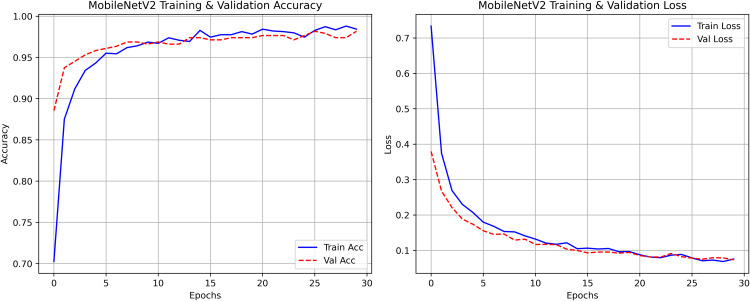
MobileNetV2 training & validation accuracy,loss.

Training accuracy of the model was 98.09 percent and a loss of 0.0757 whereas the validation accuracy stood at 98.18 percent and a loss of 0.0735 as shown in Fig 45.

**Fig 46:**
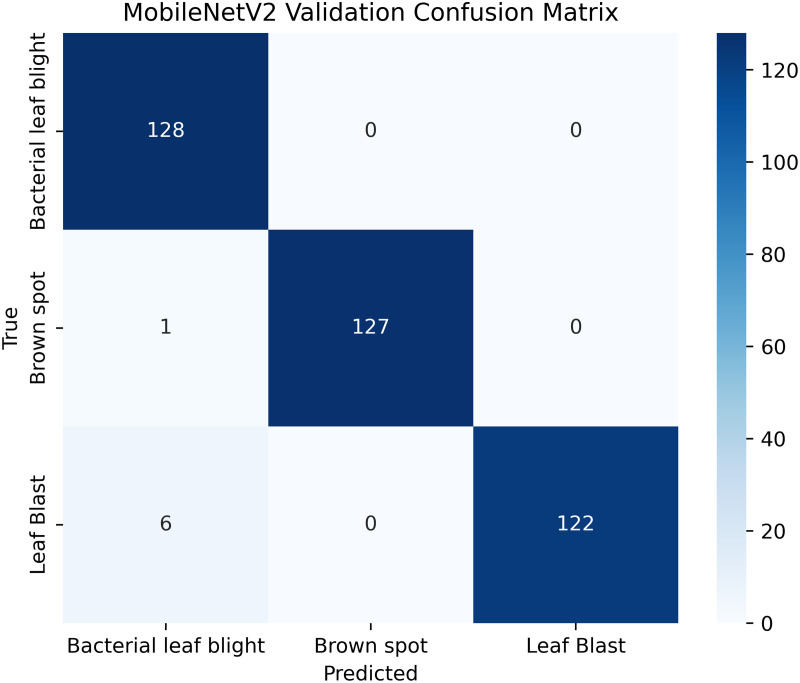
MobileNetV2 model validation confusion matrix.

The MobileNetV2 model demonstrated high accuracy by correctly identifying all 128 Bacterial leaf blight samples, while its only errors involved misclassifying 1 Brown spot case and 6 Leaf Blast cases as Bacterial leaf blight as shown in Fig 46.

**Fig 47:**
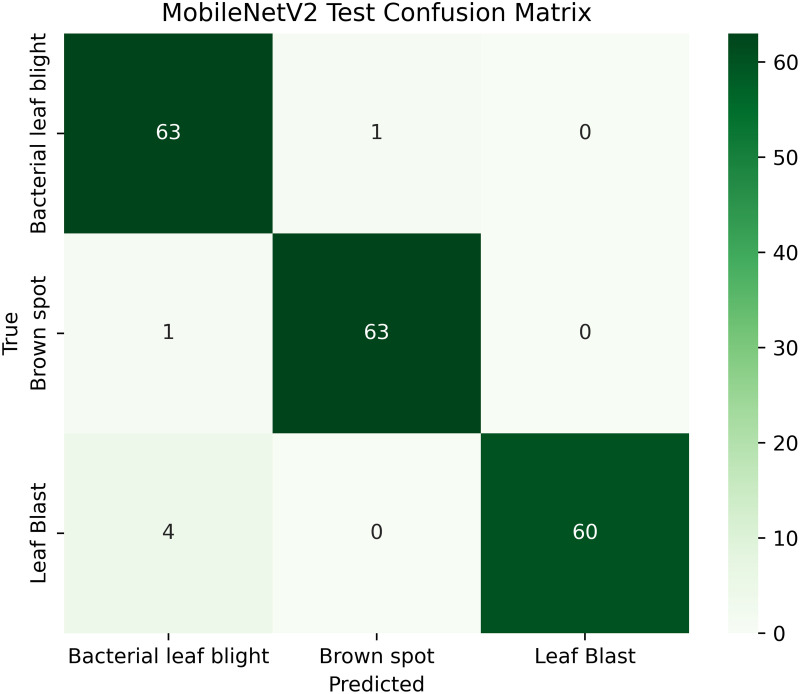
MobileNetV2 model test confusion matrix.

The MobileNetV2 test results show that 1 Bacterial leaf blight sample was misclassified as Brown spot, while 1 Brown spot sample and 4 Leaf Blast samples were incorrectly predicted as Bacterial leaf blight as shown in Fig 47.

**Fig 48:**
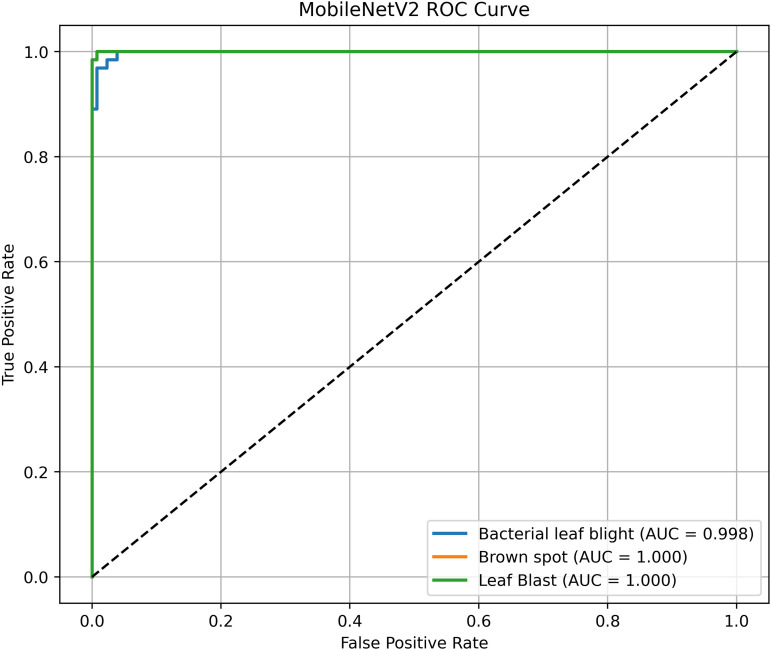
MobileNetV2 model ROC Curve (Test Set).

As the findings in the Fig 48 show, MobileNetV2 was outstanding in identifying diseases of rice leaves. With this model, the Area Under the Curve (AUC) value of the bacterial leaf blight class was found to be 0.998, which shows that this model has almost perfect discriminatory ability. In addition, it has scored an ideal AUC of 1.000 on the classes of the Brown spot and Leaf Blast. These are ideal scores that indicate that the model had the ability to fully differentiate these particular disease groups and all other categories at all thresholds, indicating perfect classification ability of the two disease types. In general, the high AUC values indicate that MobileNetV2 architecture is effective and reliable to this multi-class image classification task.

Table 9 MobileNetV2 achieved a overall test accuracy of 96.87%, demonstrating consistent performance with macro and weighted averages of 0.97 across precision, recall, and F1-score. Notably, the model attained a perfect precision of 1.00 for Leaf Blast and a balanced 0.98 across all metrics for Brown spot, while Bacterial leaf blight showed a strong recall of 0.98.

**Table 9:** MobileNetV2 Model Classification Report on Test Set.

Class	Precision	Recall	F1-score	Support
Bacterial leaf blight	0.93	0.98	0.95	64
Brown spot	0.98	0.98	0.98	64
Leaf Blast	1.00	0.94	0.97	64
**Test Accuracy**	—	—	**96.87%**	192
**macro avg**	0.97	0.97	0.97	192
**weighted avg**	0.97	0.97	0.97	192

**Fig 49:**
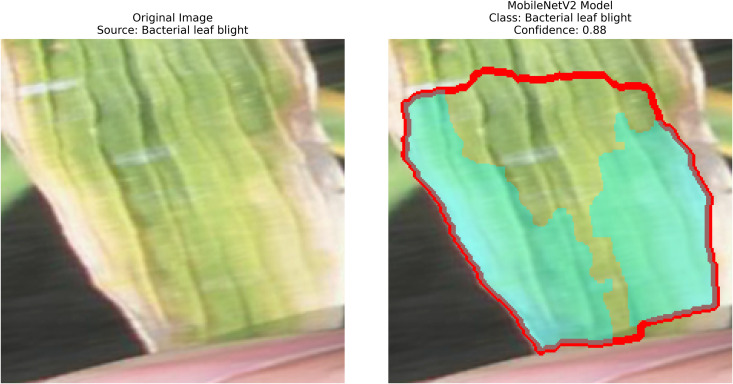
MobileNetV2 model bacterial leaf blight prediction.

**Fig 50:**
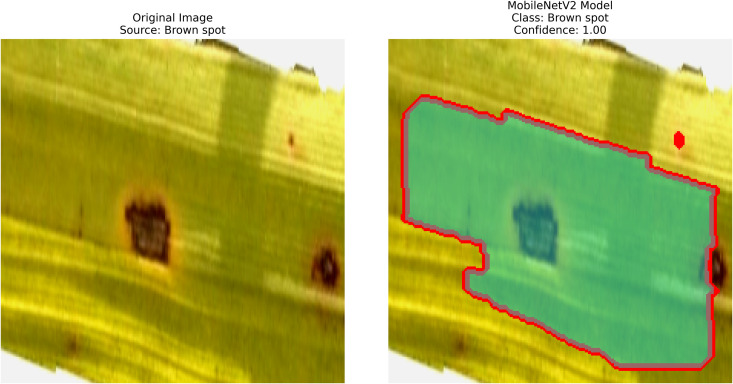
MobileNetV2 model brown spot prediction.

**Fig 51:**
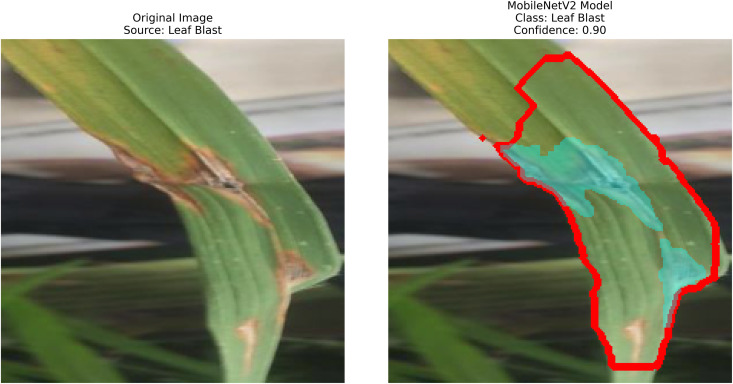
MobileNetV2 model leaf blast prediction.

Fig 49 shows an original image of a rice leaf on the left that has been correctly identified by the MobileNetV2 model as a Bacterial leaf blight image on the right with a confidence score of 0.88 which is in accord with the actual category. Fig 50 shows a picture of an original rice leaf on the left that the MobileNetV2 model has correctly identified on the right as the “Brown spot” with a perfect score (1.00) which represents the label of its original source. Fig 51 depicts a source image of a rice leaf on the left and the right hand imprints the image with the correct label of Leaf Blast with a high level of confidence score of 0.90 which correctly represents the original label.

The image of a rice leaf was used to demonstratively identify the diseased parts of the leaf based on the areas of the model (prediction confidence score). GrabCut segmentation and contour detectors were used to detect the disease parts of the rice leaf.

### Segmentation validation

Segmentation validation is a sensitive procedure in the assessment of the performance of a deep learning model in a pixel-wise classification task, e.g., detection of disease areas in rice leaf images. Segmentation metrics unlike the classification metrics that evaluate the predictions of the entire image evaluate the overlap between predicted diseased region and ground truth annotation in pixels. This paper used GrabCut segmentation and contour detection to produce disease areas and the standard metrics that were applied to evaluate the accuracy of the segmentation results against ground truth masks are as follows.

#### Intersection over Union (IoU).

Intersection over Union (IoU) or Jaccard Index is used to quantify intersection of the predicted and the actual mask of segmentation. Their intersection to their union is called their ratio:


$$IoU=\frac{TP}{TP+FP+FN}$$

- **True Positives:** Pixels that are correctly identified as belonging to the disease region.
- **False Positives:** Pixels that are incorrectly predicted as part of the disease region.
- **False Negatives:** Pixels that belong to the disease region but are mistakenly classified as background.

The values of IoU lie between 0 and 1 with the larger values implying a strong performance of segmentation. IoU is also frequently employed as a loss in deep learning models, as well as evaluation metrics in the segmentation task. In the case of rice leaf disease detection, the IoU score of over 0.5 normally implies that the region has been identified satisfactorily.

#### Dice score.

The Dice coefficient, which is also referred to as Dice score or the F1-score when applied to segmentation, is a measure of the overlap in the predicted and ground truth masks, calculated by dividing the intersection of both masks by the total number of pixels in the two masks. It is also specially sensitive to true positives, and is often used as a loss and evaluation metric in deep-learning models of segmentation because it has a stable gradient behaviour.


$$Dice=\frac{2TP}{2TP+FP+FN}$$

#### IoU & dice score result.

Table 10 is the comparison of the mean Intersection over Union (mIoU) and mean Dice (F1) scores of the various deep learning models used in disease region segmentation. The models that have been tested have the highest mIoU of 0.969 and DenseNet201 and InceptionV3 had the highest mean Dice of 0.987, which means that they have better segmentation accuracy. Contrastingly, EfficientNetV2L has much lower performance than the others, which demonstrates the efficacy of InceptionV3 and DenseNet201, in detecting the disease area accurately.

**Table 10:** Comparison of Mean IoU and Mean Dice Scores Across Different Models.

Model	Mean IoU	Mean Dice (F1)
InceptionV3	0.969	0.987
DenseNet201	0.969	0.983
ResNet152V2	0.949	0.973
EfficientNetV2L	0.283	0.430
MobileNetV2	0.940	0.967
**Best Performance**	**InceptionV3,DenseNet201**	**InceptionV3**

### Statistical significance analysis

Statistical significance analysis was conducted to identify whether the performance differences between models are statistically meaningful due to random chance. To assess whether performance differences between models are statistically significant, we use Accuracy Confedence Interval analysis and Cohen’s Kappa Statistical analysis.

#### Accuracy confedence interval analysis.

To test the statistical reliability, a 95% confidence interval was determined as follows:


$$CI=p\pm 1.96\sqrt{\frac{p(1-p)}{n}}$$

This range is a period during which the accuracy of the true model could be found within with 95 percent confidence, which assists in confirming that the observed performance is not as a result of mere chance.

Table 11 shows InceptionV3 and DenseNet201 both had a high test accuracy of 98.43% with a 95 percent confidence interval of 95.49% to 99.53%. This small range denotes high statistic reliability, implying that the actual performance of both models will always be within this range of high accuracy.

**Table 11:** Comparison of Different Models with Test Accuracy and 95% Confidence Intervals.

Model	Test Accuracy	95% Confidence Interval
InceptionV3	98.43%	[95.49%, 99.53%]
DenseNet201	98.43%	[95.49%, 99.53%]
ResNet152V2	97.39%	[93.92%, 98.97%]
EfficientNetV2L	44.50%	[37.53%, 51.70%]
MobileNetV2	96.87%	[93.22%, 98.66%]

#### Cohen’s Kappa statistical analysis.

In order to test the reliability of the classification further than just simple accuracy, Cohen Kappa coefficient was calculated using the confusion matrix of each model. Kappa, which is the measurement of agreement between predicted labels and true labels by Cohen, captures the agreement that might result simply by chance. Greater Kappa value means that there is more consistency between predictions and ground truth.

The Cohen’s Kappa statistic is defined as:


$$\kappa =\frac{p_{o}-p_{e}}{1-p_{e}}$$

Where: – *p*_*o*_ is the observed agreement (classification accuracy) – *p*_*e*_ is the expected agreement by random chance, computed from the confusion matrix

Both InceptionV3 and DenseNet201 achieve the same Cohen’s Kappa value of 0.9766,reflecting almost perfect agreement between predicted and true labels.In contrast, EfficientNetV2L records a considerably lower Kappa score (0.1719),indicates that its predictive performance is only slightly better than chance-level classification as shown in Table 12.

**Table 12:** Cohen’s Kappa Statistical Analysis for All Models on Test Set.

Model	Test Accuracy	Observed Agreement (p_o)	Cohen’s Kappa (κ)	Agreement Level
InceptionV3	98.44%	0.9844	0.9766	Almost Perfect
DenseNet201	98.44%	0.9844	0.9766	Almost Perfect
ResNet152V2	97.40%	0.9740	0.9609	Almost Perfect
EfficientNetV2L	44.79%	0.4479	0.1719	Slight
MobileNetV2	96.88%	0.9688	0.9531	Almost Perfect

### Models accuracy comparison with proposed solution

The InceptionV3, DenseNet201, ResNet152V2, EfficientNetV2L and MobileNetV2 deep learning models proved highly effective at classifying rice diseases across all training, validation, and testing phases. Table 13 shows the training, validation, and test accuracies. The results of the InceptionV3, DenseNet201, ResNet152V2,EfficientNetV2L and MobileNetV2 concerning training, validation, and test accuracy. InceptionV3 also has the highest training accuracy of 98.80% followed by validation accuracy of 98.44% and test accuracy of 98.43% which also shows the best and consistent performance across all the phases which proves that it has a high generalization ability. Despite the competitive scores of DenseNet201 (98.72%, 98.43%, 98.43%), ResNet152V2 (99.02%, 99.22%, 97.39%), EfficientNetV2L model accuracies (39.01% train, 48.70% val, 44.50% test) and MobileNetV2 model accuracies (98.09% train, 98.18% val, 96.87% test) all models had a distinct variation in validation and test performance, which indicated a slight instability relative to the highest-performing and most steady model that is InceptionV3.

**Table 13:** Model Accuracy Comparison.

Model	Train Accuracy	Validation Accuracy	Test Accuracy
InceptionV3	98.80%	98.44%	98.43%
DenseNet201	98.72%	98.43%	98.43%
ResNet152V2	99.02%	99.22%	97.39%
EfficientNetV2L	39.01%	48.70%	44.50%
MobileNetV2	98.09%	98.18%	96.87%

### Models accuracy comparison with related work

This proposed solution achieves higher accuracy levels compared to previous research by implementing advanced techniques that eliminate both overfitting and underfitting issues. The model demonstrates improved generalization and robustness, as shown by the results in Table 14, which contribute to its overall classification success. Regarding the review of various approaches, it is evident that many existing methods overlook overfitting, use minimal datasets, lack sufficient generalizability, or fall short in accuracy augmentation. Additionally, other algorithms such as InceptionV3, DenseNet201, VGG16, and EfficientNet-B0 performed well but were hindered by noisy computational demands. As compared to other related works which typically present performance differences in training and testing stages, the findings of our experimental results show that InceptionV3 shows the best balanced and stable performance among the models tested. Namely, InceptionV3 has a consistently large accuracy in training (98.80%), validation (98.44%), and testing (98.43%), hence, suggesting excellent generalization and lowering overfitting. Although DenseNet201 and ResNet152V2 reached similar levels of accuracy, their apparent performance changes indicate reduced stability, which proves InceptionV3 to be the most stable and the most efficient model in our suggested architecture.

**Table 14:** Model Performance Metrics Comparison with Related Work.

Ref.	Methodology	Dataset	Performance Metrics
[36]	InceptionV3	Rice Oryza Saitva (NA Images)	The results obtained under the classification problem using the developed model were quite accurate with a percentage of 90.77.
[37]	DenseNet201	Rice Leaf Disease (NA Images)	The model was also trained at 30, 40, and 50 epochs with the performance increasing at each stage in training; 96.09% accuracy at epoch 30, 97.71% accuracy at epoch 40 and a high performance of 100% accuracy with a small loss of 0.0352, achieved on epoch 50.
[38]	VGG16, InceptionV3 and ResNet152V2	Rice Leaf Disease (NA Images)	Simulation results reveal that VGG16, InceptionV3, and ResNet152V2 models, with selective fine-tuning, achieved 100% accuracy and an F1-score of 1.
[39]	CNNs, KNNs Classifier	Rice Classification (1045 Images)	With this method we achieved 95% accuracy in finding healthy leaf and 90% accuracy (highest with any disease) of Sheath Blight.
[40]	EfficientNet-b0, MobileNet-v2, Places365- GoogLeNet	Paddy Rice Leaf (10407 Images)	EfficientNet-b0 achieves 97.74%, MobileNet-v2 reaches 97.53%, and Places365-GoogLeNet attains 92.36% mean accuracy.
[41]	Orange 3.26	Rice Leaf Disease (120 Images)	Accuracy achieve 86.70%
[42]	MDC, KNN	Rice Leaf Disease (115 Images)	The classification of rice diseases produced the overall classification accuracy of 87.02 and 89.23 percent with the k-Nearest Neighbor (k-NN), and Minimum Distance Classifier (MDC) classifiers, respectively.
[43]	CV, SVM	Rice Leaf Disease (120 Images)	It is reported to achieve 92.5% and 87.5% of the results based on SVM and ANN classification accuracy, respectively.
[44]	LBP, HOG, SVM	Rice Leaf Disease (120 Images)	The accuracies are: LBP with Linear SVM (89.00%), Polynomial SVM (90.23%), RBF SVM (86.21%); HOG with Linear SVM (92.01%), Polynomial SVM (94.6%), RBF SVM (89.0%).
[45]	EGB Classifier	Rice Leaf Disease (120 Images)	Model achieve 86.58% Accuracy
[46]	Morphological Method, ML Algos (Bayes’ SVM)	Rice Leaf Disease (1000 Images)	79.5% Accuracy (Bayes), 68.1% Accuracy (SVM)
[47]	VGG-19 TL model	Rice Leaf Disease (2167 Images)	96.08% Accuracy, 0.9620 Precision, 0.9617 Recall, 0.9921 Specificity, 0.9616 F1-score
[1]	SqueezeNet	Rice Leaf Disease (2250 Images)	99.30% accuracy, 0.972–1.000 precision, 0.980–1.000 recall, 0.0–0.3% error rate on rice disease classification.
[2]	Hybrid CNN-VGG Deep Learning model	Rice Leaf Disease (1800 Images)	Model achieve 95.60% Accuracy
	**InceptionV3, DenseNet201, ResNet152V2, EfficientNetV2L and MobileNetV2**	**Rice Leaf Disease (1914 Images)**	**InceptionV3 had the most consistent and stable performance with 98.80% training, 98.44% validation, and 98.43% test accuracy, having better generalization, but ResNet152V2 and DenseNet201 though also very competitive, had more significant variances of validation-test accuracy which suggests lower stability and showed noticeable fluctuations during training in graph. EfficientNetV2L model demonstrated comparatively poor performance in this experimental setting, while MobileNetV2 model still maintained reliable classification capability despite being a lightweight architecture.**

Our method employs GrabCut segmentation and active contour estimation to provide tightly localized and accurate disease detection. Unlike previous attempts, we effectively manage both overfitting and underfitting issues. The model also shows strong generalization across different subspecies of the disease, even those with fewer resources. Overall, the proposed approach is reliable, fast, and efficient compared to existing methods in the surveyed literature. The integration of GrabCut segmentation with contour detection algorithms is crucial in achieving these improvements. The accuracy of input data increases when diseased areas are precisely separated using GrabCut segmentation technology. Additionally, contour detection enhances rice leaf disease identification by highlighting essential features that enable the system to better distinguish subtle visual elements. This independent comparative evaluation enhances entire model performance and enables precise diagnosis of rice leaf diseases.

## Cross-dataset generalization analysis

**Fig 52:**
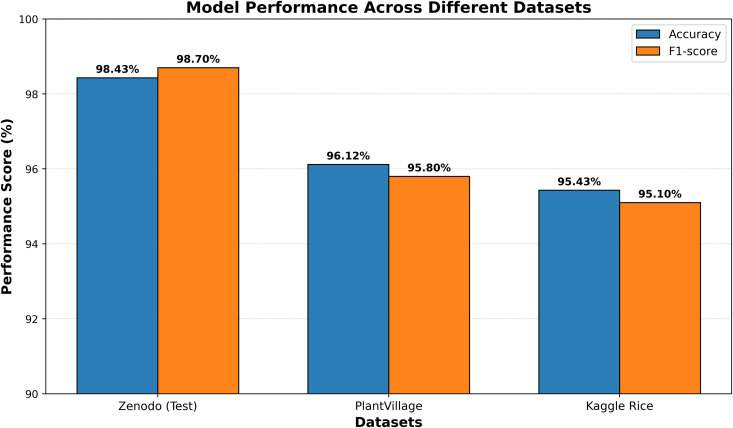
Model performance across different datasets.

The model performance across different datasets shows the highest accuracy and F1-score on the Zenodo (Test) set at 98.43% and 98.70% respectively, followed by 96.12% accuracy on PlantVillage and 95.43% on Kaggle Rice as shown in Fig 52. The moderate decrease in performance reflects domain shift due to lighting, background, and acquisition differences. However, the model maintains strong generalization ability. The proposed model achieves the highest performance on the Zenodo test dataset, attaining 98.43% accuracy and 98.7% F1-score, demonstrating its strong capability for accurate rice leaf disease classification as shown in Table 15.

**Table 15:** Performance Metrics Across Different Datasets.

Dataset	Accuracy	F1-score
Zenodo (Test)	98.43%	98.70%
PlantVillage	96.12%	95.80%
Kaggle Rice	95.43%	95.10%

## Repeated experimental validation

Each model was trained four times using different random seeds (42, 100, 202, 512). Performance metrics were average. Low standard deviation confirms training stability and robustness. Most models have very small standard deviation values, meaning that their classification performance is highly stable when using different random seeds, but the relatively large deviation of EfficientNetV2L suggests higher variance and the lack of learning stability during the training process as shown in Table 16.

**Table 16:** Performance Statistics Across Four Random Seeds.

Model	Mean Accuracy (%)	Std. Dev	Mean F1-score (%)
InceptionV3	98.36	± 0.05	98.63
DenseNet201	98.35	± 0.06	98.22
ResNet152V2	97.33	± 0.04	97.24
EfficientNetV2L	38.62	± 0.31	42.46
MobileNetV2	98.03	± 0.05	96.62

## Discussion

### Theoretical contributions

This research has three main contributions to literature. First, it offers statistically proven comparative comparison against various deep transfer learning architectures of rice leaf disease. Second, it explains the purpose of post hoc segmentation as an interpretability mechanism and not a preprocessing step to improve performance, thus eliminating the methodological confusion of previous. Third, inclusion of statistical significance testing, repeated experimental execution and cross-dataset validation helps this study to enhance methodological rigor in the research on agricultural deep learning. This work, in contrast to many others that emphasized accuracy reporting, is motivated by reproducibility, statistical validation, and explainable AI integration.

### Practical implications

Practically, the proposed framework has a number of benefits to real-world agricultural implementation. Firstly, high classification accuracy and localization visualization allows agronomists to detect regions with diseases effectively. Second, transfer learning is less expensive in terms of computational cost and training time than fully trained deep models. The interpretability of GrabCut-guided segmentation with contour detection can be helpful in boosting the confidence of agricultural players since the predictions are visually correlated with the areas of influence.

## Limitations

Although the proposed framework demonstrates high classification and localization performance, several limitations should be acknowledged. A limitation of this experiment is that the DenseNet201 and ResNet152V2 models had intermittent and relatively volatile training dynamics, with some inconsistency in the convergence patterns between epochs. EfficientNetV2L exhibited suboptimal training behavior, with low accuracy and elevated loss values across both training and validation phases, achieving relatively low training accuracy 39.01%, validation accuracy 48.70%, and test accuracy 44.50% along with high loss values, suggesting insufficient convergence and poor generalization during training.

## Conclusion

Advanced deep learning combined with image processing techniques enables the automatic improvement of early rice crop disease detection accuracy. In this study, we aimed to develop an efficient method for detecting and classifying Bacterial Leaf Blight, Brown Spot, and Leaf Blast using pre-trained transfer learning models InceptionV3, DenseNet201,ResNet152V2, EfficientNetV2L and MobileNetV2, integrated with image processing techniques such as GrabCut segmentation and contour detection.

The research presents state-of-the-art methods for early detection of rice diseases through the integration of deep learning models and image processing techniques. It focuses on accurately classifying three major rice leaf diseases: Bacterial Leaf Blight, Brown Spot, and Leaf Blast, utilizing pre-trained models InceptionV3, DenseNet201,ResNet152V2, EfficientNetV2L and MobileNetV2. InceptionV3 was found to be the most stable and high-performing of the evaluated architectures, with a peak training accuracy of 98.80, both validation and test accuracy of 98.44 and 98.43, respectively, which implies good stability and a high generalization capacity. Comparatively, DenseNet201 (98.72%, 98.43%, 98.43%), ResNet152V2 (99.02%, 99.22%, 97.39%), EfficientNetV2L model accuracies (39.01% train, 48.70% val, 44.50% test) and MobileNetV2 model accuracies (98.09% train, 98.18% val, 96.87% test) were performing competitively but had significant variations between test and validation.These results indicate that InceptionV3, exhibits the strongest generalization ability and consistent performance in classifying rice diseases.

To enhance classification accuracy, the study incorporated GrabCut segmentation and contour detection algorithms to precisely localize and visualize the infected areas of the leaves. The interaction between segmentation and classification is designed so that InceptionV3, DenseNet201,ResNet152V2, EfficientNetV2L and MobileNetV2 serve as fundamental models in disease categorization. The predicted outputs are then processed with GrabCut and contour detection techniques to localize and highlight diseased regions. This post-processing step improves model interpretability by providing visual evidence of the decision-making regions. This approach addresses a critical challenge in key rice-producing nations like Pakistan by automating disease detection and eliminating the inefficiencies of manual monitoring. Overall, the research underscores the benefits of combining artificial intelligence with digital image processing (DIP), resulting in a simple, accurate, and reliable rice disease detection system.

Recent studies (2023–2026) report classification accuracies between 95% and 98% using transfer learning architectures such as EfficientNet, VGG-based hybrids, and Vision Transformer variants. While comparable performance levels have been achieved in controlled datasets, many studies lack rigorous statistical validation and interpretability analysis. In contrast, the present study demonstrates statistically significant improvements over classical CNN backbones while integrating explainable localization through GradCut segmentation. Additionally, cross-dataset validation confirms generalization capacity under domain shift conditions, strengthening practical reliability beyond isolated benchmark performance.

Future research in rice leaf disease classification will explore using drones for real-time field data collection, enabling the mapping of large agricultural areas over time. High-resolution aerial images will be analyzed with advanced computer vision algorithms to detect early, subtle disease signs and to differentiate various disease stages across different environments through multi-spectral imaging. Our method will leverage deep learning models to automate and accurately identify and categorize diseases. Currently, the model is limited to per-image analysis, which does not consider the disease’s progression over time, and struggles with the effectiveness of GrabCut segmentation in complex field textures and varying light conditions. Additionally, models are not adjustable in real time and perform poorly on rare or early-stage disease cases. Moving forward, multi-temporal and multi-spectral images captured by drones will be integrated into systems that support dynamic data monitoring, including online learning techniques. We will also develop a real-time system using Streamlit to offer interactive visual analytics and timely decision support in precision agriculture. A robust web application built with Python and Streamlit will visualize live data streams and feature interactive dashboards for easy monitoring. Real-time alerts and detailed analytics will enable farmers to make timely, informed decisions to protect their crops. In conclusion, this integrated approach emphasizes the potential of precision agriculture to promote sustainable rice farming practices.
