# Correction: Enhanced rice leaf disease classification via contour-driven segmentation and optimized deep transfer learning architectures

**DOI:** 10.1371/journal.pone.0352097

**Published:** 2026-06-17

**Authors:** Ummer Shakeel, Muhammad Asif Habib, Muhammad Yasir, Muhammad Umar Chaudhry, Muhammad Munwar Iqbal, Mudassar Ahmad, Qaisar Abbas, Muhammad Abdul Qayum

There are errors in the Funding statement. The correct Funding statement is as follows: This work was supported and funded by the Deanship of Scientific Research at Imam Mohammad Ibn Saud Islamic University (IMSIU) (grant number IMSIU-DDRSP2602).
